# Efficient traffic sign recognition using YOLO for intelligent transport systems

**DOI:** 10.1038/s41598-025-98111-y

**Published:** 2025-04-21

**Authors:** Cong Wang, Bin Zheng, Chenxing Li

**Affiliations:** 1 School of Information and Electrical Engineering, Panzhihua University, Panzhihua, 617000 China; 2 School of Intelligent Manufacturing, Panzhihua University, Panzhihua, 617000 China; 3 School of Mathematics and Computers Big Data Science, Panzhihua University, Panzhihua, 617000 China

**Keywords:** Traffic sign recognition, Deep learning, Target detection, Mechanical engineering, Information technology

## Abstract

Accurate traffic sign recognition (TSR) is critical for enhancing the safety and reliability of autonomous driving systems. This study proposes an optimized YOLOv5-based framework to address challenges such as small-scale detection, environmental variability, and real-time processing constraints. Three key innovations are introduced: (1) k-means++ clustering for anchor box optimization, achieving a 77.55% average IoU (vs. 75.95% for traditional k-means) to enhance small-target detection; (2) comprehensive comparative analysis of YOLOv5 variants (s/m/x), revealing precision-speed trade-offs (99.3–99.5% mAP@0.5 vs. 32–45 ms inference time) for deployment flexibility; and (3) systematic hyperparameter tuning to maximize robustness across diverse scenarios. Leveraging the CCTSDB dataset (13,830 annotated images), experiments demonstrate the framework’s superiority: it attains 98.1% mean average precision (mAP), 98.6% recall, and 99.3% precision, outperforming Faster-RCNN and SSD by 5–8% in mAP while maintaining 45 FPS throughput. The YOLOv5s variant achieves optimal balance with 99.3% mAP@0.5 and 32 ms per-image inference, validated through rigorous statistical analysis (Tukey HSD). Robust performance in challenging conditions (e.g., small sample, backlit sample, foggy scenes) is evidenced by detection confidence exceeding 0.90. These results highlight the framework’s applicability in latency-sensitive intelligent transportation systems.

**Supplementary Information:**

The online version contains supplementary material available at 10.1038/s41598-025-98111-y.

## Introduction

Intelligent Transportation Systems (ITS) have become a cornerstone of modern urban development, with autonomous driving technologies playing a pivotal role in enhancing road safety and efficiency. A critical component of autonomous systems is accurate Traffic Sign Recognition (TSR), which provides real-time navigational guidance and regulatory compliance for vehicles. However, TSR faces significant challenges due to small object sizes, environmental variations (e.g., lighting, weather), and real-time processing constraints. This study addresses these challenges by proposing an optimized YOLOv5-based framework, leveraging anchor box optimization, systematic model variant analysis, and hyperparameter tuning to achieve robust and efficient TSR performance.

Traffic signs are essential for safe driving, conveying critical information such as speed limits, prohibitions, and directions. Traditional TSR methods, relying on manual feature extraction (e.g., color-based or shape-based approaches), struggle with complex scenarios due to limited feature representation capabilities and computational inefficiencies^1–3^. For instance, color segmentation in RGB space is prone to background noise^4^, while shape-based methods using HOG or SIFT often require extensive computational resources^5,6^. These limitations necessitate advanced solutions capable of handling dynamic environments and real-time demands.

Deep learning, particularly Convolutional Neural Networks (CNNs), has revolutionized TSR by enabling automatic feature learning. Among CNN-based approaches, the YOLO (You Only Look Once) series has emerged as a leader in real-time object detection. Recent advancements in YOLO variants include hierarchical detection frameworks and spatial feature enhancement techniques. For example, Usmani et al.^7^ proposed a hierarchical YOLO with real-time text recognition for bilingual (Arabic-English) traffic signs, achieving 0.9124 mAP and 89.3% word accuracy. However, this approach focuses on text recognition rather than small-target detection or environmental robustness. Similarly, Zhang et al.^8^ introduced a pixel-wise spatial feature enhancement module (PSFE-YOLO) to balance computational efficiency and spatial attention, but their method was validated on specific datasets (GTSDB, TT100K) and may not generalize to diverse scenarios.

Despite these innovations, three key limitations persist in YOLO-based TSR:

Small target detection: Traffic signs often occupy < 1% of the image area, requiring specialized anchor box design. Existing methods like PSFE-YOLO^8^ focus on spatial feature enhancement but lack anchor optimization tailored to small objects^9^.

Environmental robustness: Performance degradation under challenging conditions (e.g., backlighting, fog) remains a concern. Most studies, including hierarchical YOLO^7^, do not systematically address environmental variability^10^.

Deployment flexibility: A lack of systematic analysis on model variant selection (e.g., YOLOv5s/m/x) hinders optimal deployment across hardware platforms. Current works, such as traffic signal control using YOLO^11^, prioritize application-specific optimization over generalizable model selection frameworks^12^.

To address these gaps, this research introduces three innovations:

Anchor box optimization: A k-means++ clustering algorithm is applied to generate anchor boxes tailored to the CCTSDB dataset^13^, improving small-target detection with a 77.55% average IoU (vs. 75.95% for traditional k-means)^14^. This addresses the limitation of generic anchor boxes used in previous studies like^7,8^.

Model variant analysis: A comparative evaluation of YOLOv5s/m/x variants reveals precision-speed trade-offs (99.3–99.5% mAP@0.5 vs. 32–45 ms inference time)^15^, guiding deployment decisions. This systematic analysis fills the gap in deployment flexibility identified in^11^.

Hyperparameter tuning: A systematic optimization strategy enhances model robustness across diverse scenarios, validated through rigorous statistical analysis (Tukey HSD)^16^. This approach complements spatial feature enhancement methods^8^ by ensuring stability in challenging environments^12^.

The CCTSDB dataset (13,830 annotated images) is employed, covering 138 Chinese traffic sign categories^17^. Data augmentation (Mosaic) and transfer learning are integrated to improve generalization^18^. The proposed framework is evaluated against Faster R-CNN, SSD, and YOLOv5 variants, demonstrating superior performance: 98.1% mAP, 98.6% recall, and 45 FPS throughput^19^.

## Theoretical overview and image preprocessing based on the YOLOv5

**Fig. 1:**
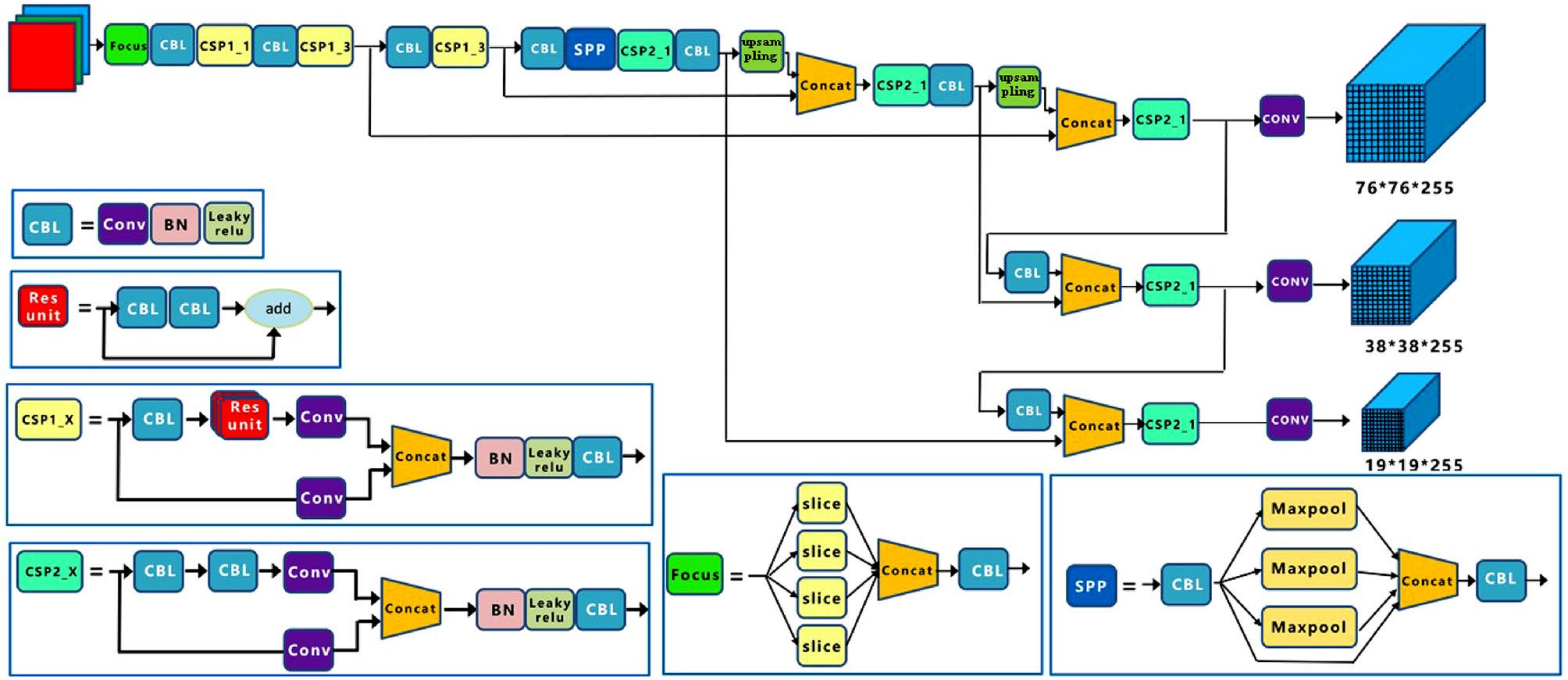
Schematic of YOLOv5 network.

Compared with the R-CNN series algorithm, the YOLOv5 series algorithm can realize end-to-end processing and output of data, obtaining a better detection speed. Figure 1 gives the YOLOv5 network structure. This paper uses the YOLOv5 model, which has four model structures. They are YOLOv5s, YOLOv5m, YOLOv5l, and YOLOv5x. YOLO5s is the smallest and fastest one, but its detection accuracy is the lowest^20^. The other models, YOLOv5m, YOLOv5l, and YOLOv5x,widen and deepen their network structure based on YOLOv5s in turn to improve the detection accuracy, but their speeds decreases relatively.

### Image augmentation and backbone structure

Yolov5 follows the Mosaic data enhancement method of YOLOv4. That is, several images are randomly selected, and put several images together into one image for data training. Meanwhile, the image color gamut is changed by flipping the image.

In the YOLO algorithm, for different kinds of data sets, the corresponding anchor widths of various lengths and widths will be set. At the same time, a prediction box will be output based on the original bounding box, so that the prediction box can be compared with the actual box^21^. The parameters can be continuously optimized through repeated comparisons.

**Fig. 2:**
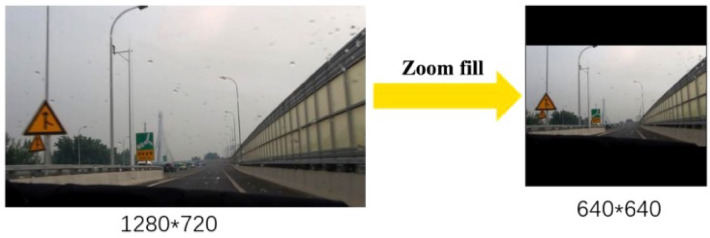
Zoom fill.

For object detection algorithms, in the case that the length and width of images are different, all original images can be scaled to a specified size for subsequent detection. Figure 2 shows the scaling of an image with 1280*720 to image 640*640. Thus, the amount of calculation is reduced during processing, and the detection speed is improved.

In the actual operation process, images with different aspect ratios have different sizes of black edges at both ends after scaling and filling. In particular, the side with a larger black edge will lead to information redundancy and slow the speed of speculation. To address this, YOLOv5 implements a dynamic scaling strategy in its preprocessing pipeline, which adaptively calculates the optimal zoom ratio for each image and applies minimal asymmetric padding. This operation is divided into three steps.

The original zoom size is 640*640. Divide the dimensions of the original image by the target size to obtain two zoom coefficients of 0.5 and 0.89. Then, the smaller coefficient is selected as the subsequent zoom coefficient. The length and width of the original image are multiplied by the zoom coefficient selected in the first step of 0.5, and the length becomes 640, and the width becomes 360. Take the difference of 280 between 640 and 360 to obtain the height of the image to be filled, and 32 is substracted from this number to get 24 pixels, and then get the value of the image width filled. At this time, divide the value obtained above by 2 to get the height of the padding at both ends.

The Backbone part mainly consists of Focus subsampling and Cross Stage Partial connections (CSP). The function of Focus subsampling is to reduce the dimension of features while retaining the practical information needed by the models. Therefore, avoiding over-fitting to a certain extent is simply a way to reduce the image^22^. However, these methods are inevitably at the cost of sacrificing part of the information, relying on reducing the amount of data, making the amount of calculation required less. Down-sampling is similar to the pooling operation. But the difference between pooling and it is that pooling also needs to consider rotation, translation, and other interferences. There are maximum sampling methods, average sampling methods, and random sampling methods. The idea of CSP is to divide the feature map into two parts according to channels, one part is processed by a series of convolutional layers. The other part is directly processed for the next step of calculation. These two parts are merged through a residual connection after processing^23^. It can provide higher-level features, accelerate the speed of feature extraction, reduce the number of model parameters, and thus enable YOLOv5 to perform better object detection.

Two CSP structures are used in YOLOv5, namely, CSP1_X and CSP2_X. Where, CSP1_X is used for the Backbone network, and CSP2_X is used for the Neck. In the reasoning process of the CSPNET, the repeated computation caused by repeated gradient information is excessive computation. Hence, in the CSP model, feature mapping of its basic level is divided into two independent parts. The cross-stage hierarchy is used to combine them to ensure accuracy, decrease computation, and increase speed.

### Neck structure

In the framework of target detection network structure, the neck structure plays an essential role in transition. It can not only conducive to the specific task learning of the head in the next step but also process and utilize the crucial features extracted from the backbone in the previous step. The neck of YOLOv5 follows YOLOv4 and adopts the FPN + PAN structure.

**Fig. 3:**
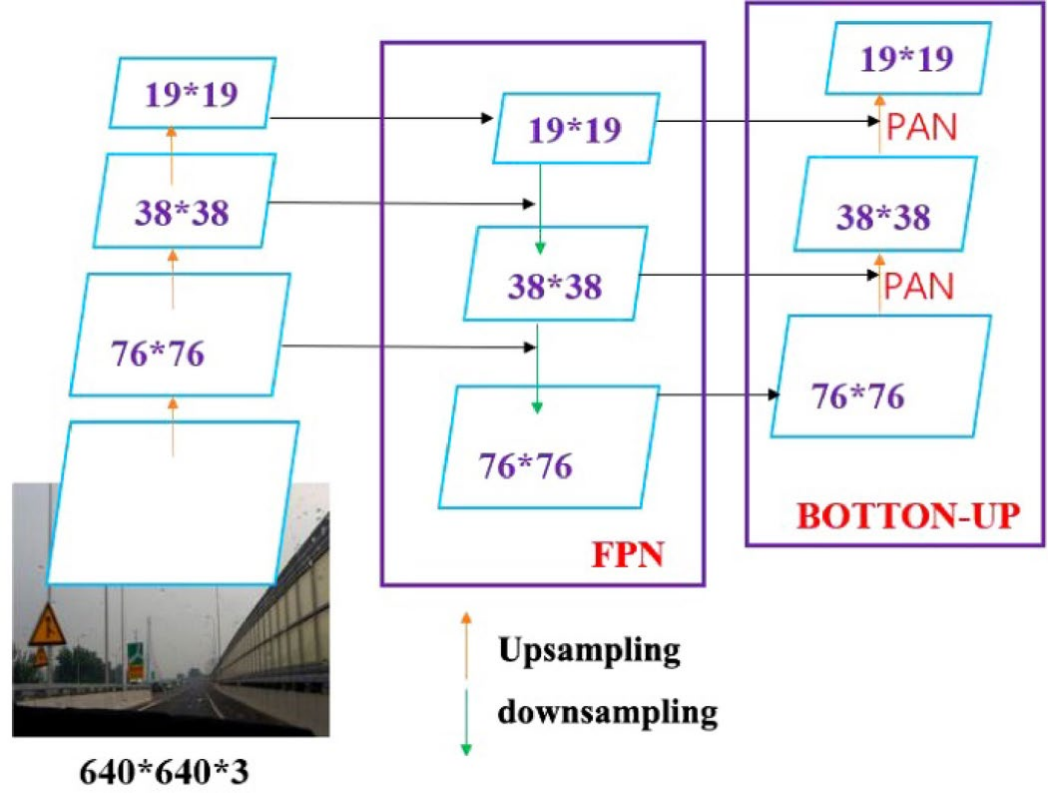
The processing flow of the FPN + PAN feature pyramid.

The FPN is used to up-sample the deep-seated feature map and combine it with the shallow network to transfer it from high to low to achieve the fusion of semantic information and detailed information of different scales. PAN aims at this point by adding a reverse channel behind the FPN to supplement the FPN and transmit the low-level information, as shown in Fig. 3.

However, YOLOv5 also has some optimization parts. YOLOv5 abandons the general convolution calculation operation previously used by YOLOv4, and it uses the CSP2 structure in CSPNET structure design to make its neural network feature fusion more powerful.

### Output—loss function

There are two categories in the Output-loss function. One is composed of the classification loss function. Specifically, the current classification function still adopts BCE loss. The other is the regression loss function, whose development history is from smooth L1 Loss to IoU Loss (2016), then GIoU Loss (2019), then DIoU Loss (2020), and finally CIoU Loss (2020).

**Fig. 4:**
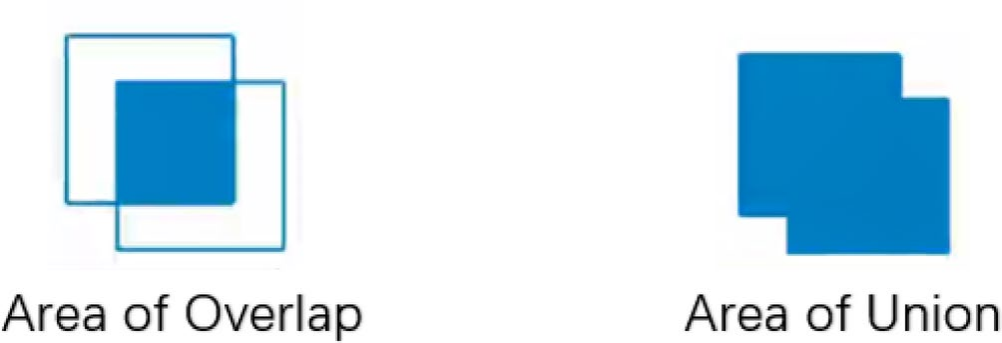
Demonstration diagram of IoU loss.

Intersection over Union (IoU) is used to describe the accuracy of prediction boxes. IoU is an important indicator in target detection problems, which reflects the degree of overlap between the annotated box and the predicted one during the training phase, as shown in Fig. 4.

1 $$IoU = \frac{Area\;of\;Overlap}{{Area\;of\;Union}}$$

GIoU is the IoU subtracting the proportion of the part of the external frame that does not belong to the intersection of the actual one and the prediction frame with the external frame, as shown in formula (2).

2 $$GIoU = IoU - \frac{{\left| {B_{C} - U} \right|}}{{\left| {B_{C} } \right|}}$$

**Fig. 5:**
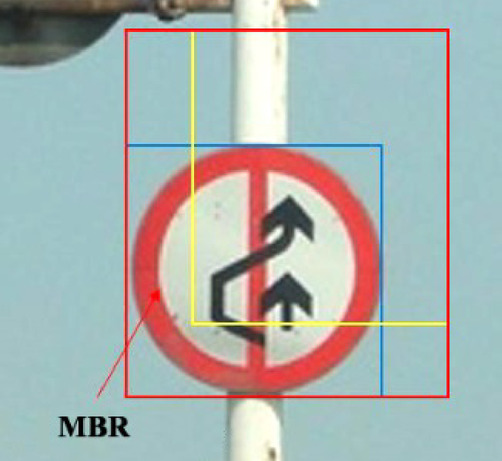
External block.

**Fig. 6:**
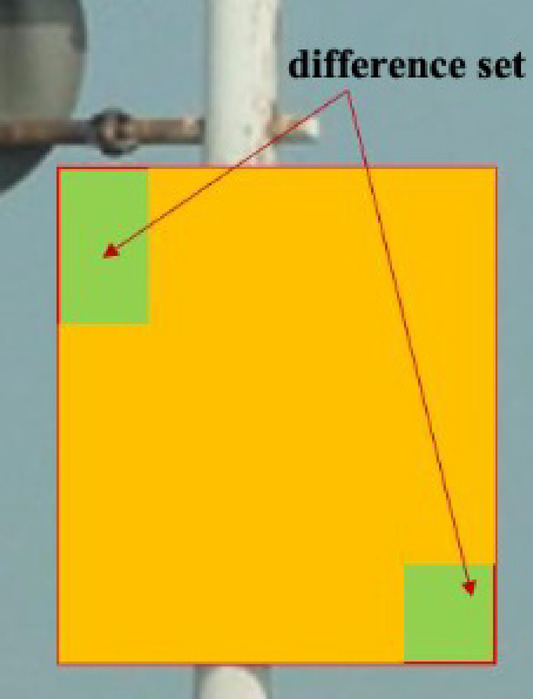
Difference set.

GIoU can be understood in Figs. 5 and 6.

The area of the circumscribed box of the two boxes = the red rectangular area, IoU = the intersection/union of the yellow box and the blue one, and specific gravity = the green area/the red matrix area.

**Fig. 7:**
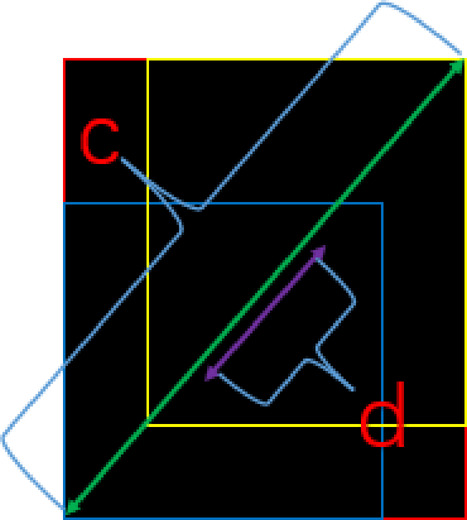
Demonstration diagram.

DIoU is optimized and improved based on GIoU, in which the distance between the target and anchor, overlap rate, and scale are added. Therefore, its target box regression mechanism becomes more stable than GIoU.

3 $$DIoU = IoU - \frac{{d^{2} (a,a^{gt} )}}{{c^{2} }}$$

where ***c*** refers to the diagonal distance between the outer frame zone of the prediction frame and the actual frame, and ***d*** refers to the distance between the two center points. *ɑ* and *ɑ*^*gt*^ refer to the center of prediction and the actual box, respectively. It is shown in Fig. 7.

The target box regression loss requires attention to the following three elements, overlapping area, center distance, and aspect ratio. It is also essential to consider the consistency of the aspect ratio between the anchor box and the target one. Hence, Complete-IoU (CIoU) loss method is used, as shown in formula (4).

4 $$CIoU = 1 - IoU + \rho^{2} \frac{{(b,b^{gt} )}}{{c^{2} }} + av$$

where α is a weight function, and v is used to calculate the aspect ratio similarity. It is defined as shown in Eq. (5).

5 $$\begin{aligned} v & = \frac{4}{\pi }\left( {\arctan \frac{{w^{gt} }}{{h^{gt} }} - \arctan \frac{\omega }{h}} \right)^{2} \\ \alpha & = \frac{\nu }{{\left( {1 - IoU} \right) + \nu }} \\ \end{aligned}$$

## Experiment and test

This section mainly introduces the training process of the model from the perspective of data set production and division, hardware, and software environment layout. It carries out relevant parameter adjustment and index evaluation to obtain the optimal solution to realize traffic sign recognition.

### Construction of data set

The experimental environment is as follows. The operating system is Windows 10, the graphics card (GPU) is GEFORCE GTX 3060, the video memory is 12 GB, and the processor is Intel ® Core ™ I7-12700F. The CCTSDB is the data set. It contains about 16,000 images, and its labels are divided into three classifications. They are warning, prohibition, and mandatory signs. This data set includes the most common traffic signs, such as lighting ways, diversity of shooting angles, different time backgrounds, etc. In general, to train a good prediction model, the larger the data set, the better the training effect. Based on the conditions of the experiment system and the related environment, 13,830 images were selected in this training.

**Fig. 8:**
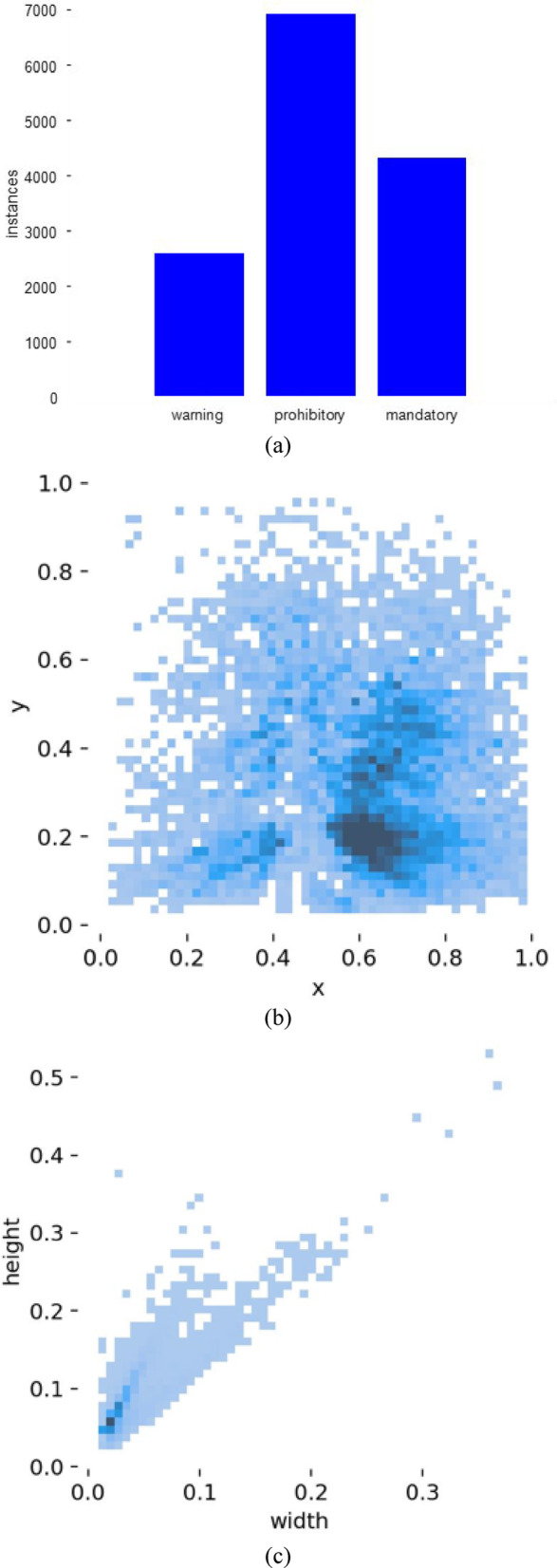

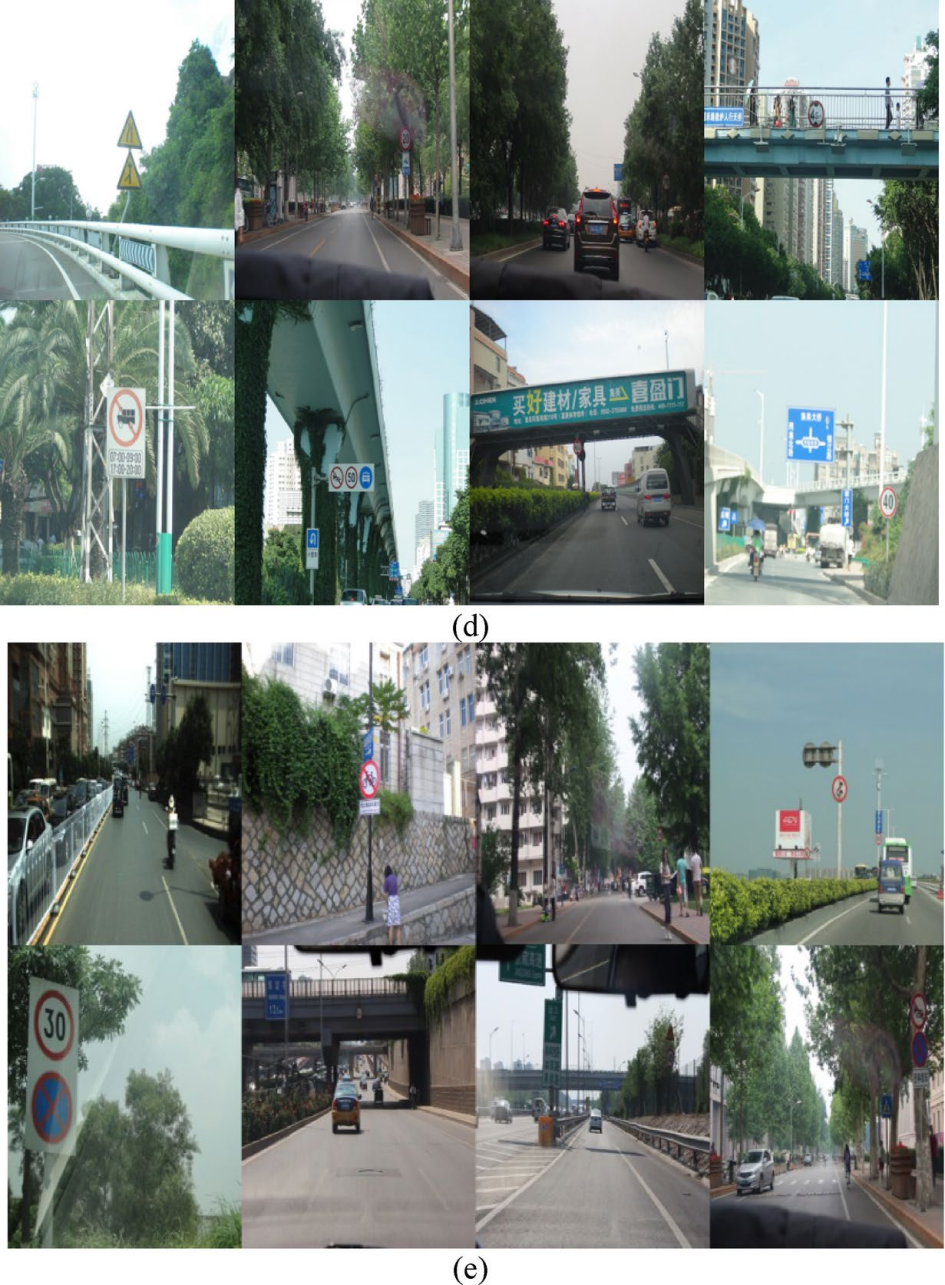
Data set. (**a**) Bar graph of the number of targets of each type. (**b**) Normalized target location diagram. (**c**) Normalized target size diagram. (**d**) Partial image of the training set. (**e**) Partial images of the verification set.

Figure 8a shows the statistical data of the target number in each category divided into warnings, prohibition, and mandatory signs.

The following measures have been taken to address the imbalance issue of three types of sign data (2593 warning signs, 6915 prohibition signs, and 4322 mandatory signs). The k-means clustering algorithm is used to optimize anchor boxes, enabling the model to better adapt to the size of different category labels. The Mosaic technology is used to stitch images of different categories. This further enriches the diversity of training data and helps the model learn more robust features. These measures effectively alleviate the problem of data imbalance and improve the detection performance of the YOLO model.

Figure 8b shows the normalized target location map. The coordinate origin is set in the lower left corner of the dataset image, and the values of the X-axis and Y-axis are relative coordinate values used to evaluate the relative position of the target image. From the figure, the target image is distributed across a wide range of the coordinate system, but is mainly concentrated in the lower right corner of the coordinate system, which is relatively concentrated on the X-axis and relatively dispersed in the Y-axis direction. Figure 8c is a normalized target size map. It can be seen from the figure that the target size distribution is concentrated. The width and height are distributed about zero to one point five, and the target size is relatively small. To train the data set in YOLOv5 and keep consistent, the XML file is randomly divided into a training set and a verification one at 8:2. One part of the training set for traffic sign target detection and positioning is shown in Fig. 8d. The other part of the verification set is shown in Fig. 8e.

As shown in Fig. 8d, combined with the characteristics of the CCTSDB data set structure, 10,074 images with different shooting times, light, angles, backgrounds, and scenes are chosen as the training sets for traffic sign recognition. Figure 8e shows the remaining 2766 images under the same conditions as the verification sets for traffic sign recognition.

### k-means++ algorithm clustering data set

To increase traffic sign detection accuracy, the k-means++ clustering algorithm is devoted to clustering the memo data set. It is used to determine the optimal prior frame by having a large intersection between the anchor frame and the detection one, as shown in formula (6).

6 $$d = 1 - IOU$$

Here, IOU refers to the intersection ratio of the predicted and actual frame. It is allocated based on the principle of small-scale large a priori box and large-scale small a priori box.

**Fig. 9:**
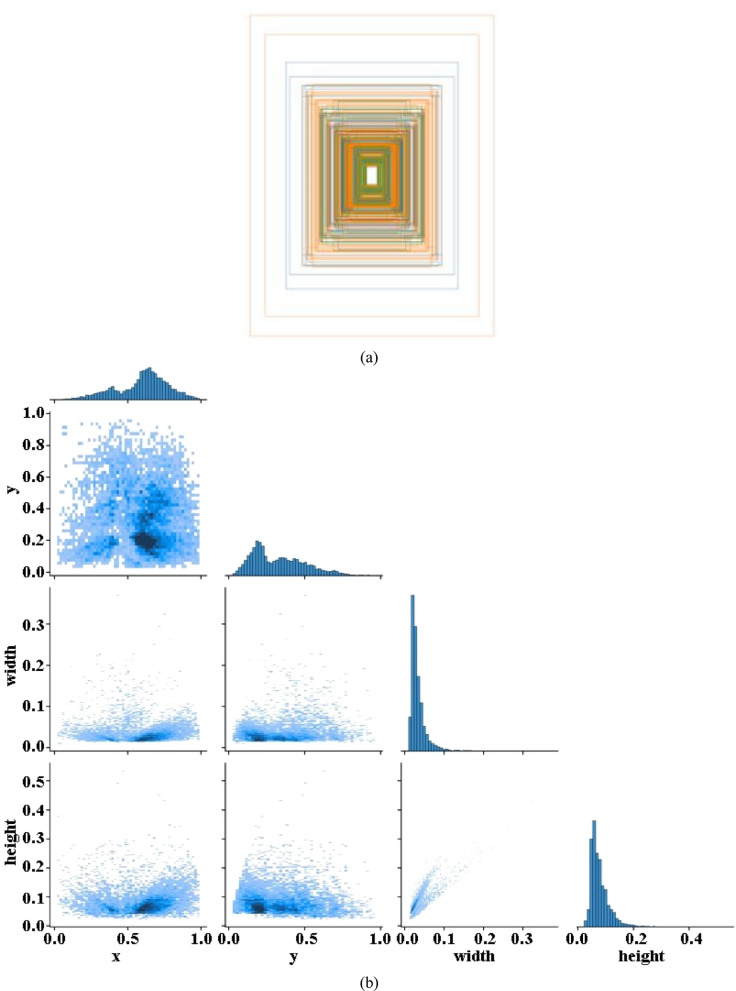

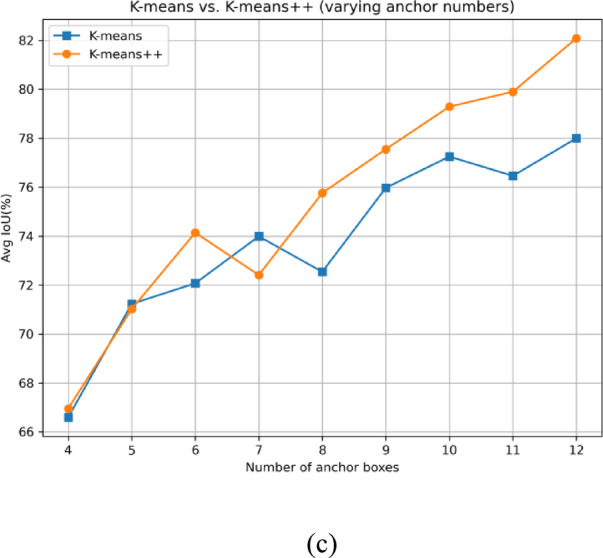
(**a**) Traffic sign tracing block diagram. (**b**) k-means++ cluster anchor distribution diagram. (**c**) Comparison between k-means and k-means++.

Figure 9a is an anchor frame distribution diagram showing the intuitive situation of the data label. By comprehensively analyzing the data on the label of the target position and size, the target relative position map and the target relative size map are obtained, as shown in Fig. 9b.

Figure 9b shows the establishment of a rectangular equation coordinate system with the lower left corner of the image as the coordinate origin. According to this, we find that the traffic sign anchor box are concentrated in the range of 0.5–0.8 along the X-axis coordinate direction and 0.0–0.2 along the Y-axis coordinate direction. The target width mainly accounts for 3–13%, and the target height mainly accounts for 4–15%. From the above analysis, it can be seen that there is a considerable gap between the distribution of candidate frames in the initial region and the data set. The reason is that the target sample data set includes objects of different sizes, resulting in insufficient detection of small targets.

The traditional k-means algorithm randomly selects the initial center, which may result in uneven distribution of initial centers or multiple centers crowded together, leading to slow convergence or even convergence to local optima. k-means++ algorithm selects new cluster centers in a probability distribution by calculating the distance from each sample to the selected center, making the initial center distribution more reasonable, thereby reducing unnecessary iterations and shortening clustering time. In the case where YOLOv5 requires 9 anchor boxes, k-means++ only took 14 iterations, while standard k-means requires 51 iterations. The average IoU of k-means++ is 77.55%, and the average IoU of standard k-means is 75.95%. The use of k-means++ not only reduces the computational complexity of the clustering stage but also makes the model converge easily in subsequent training, thereby improving the overall training speed. Therefore, using k-means++ in the preprocessing of YOLOv5 can reduce repeated iterations, accelerate convergence, and obtain better anchor box initialization during anchor box clustering, thereby improving overall computational efficiency and detection performance to a certain extent. The comparison image is shown in Fig. 9c.

According to experimental results, using the k-means++ algorithm can effectively decrease the time of the model searching for candidate boxes and the amount of calculation in the process of actual box matching and set the appropriate anchor to carry out the task of traffic sign recognition. It has achieved good results.

### Image input and preprocessing

**Fig. 10:**
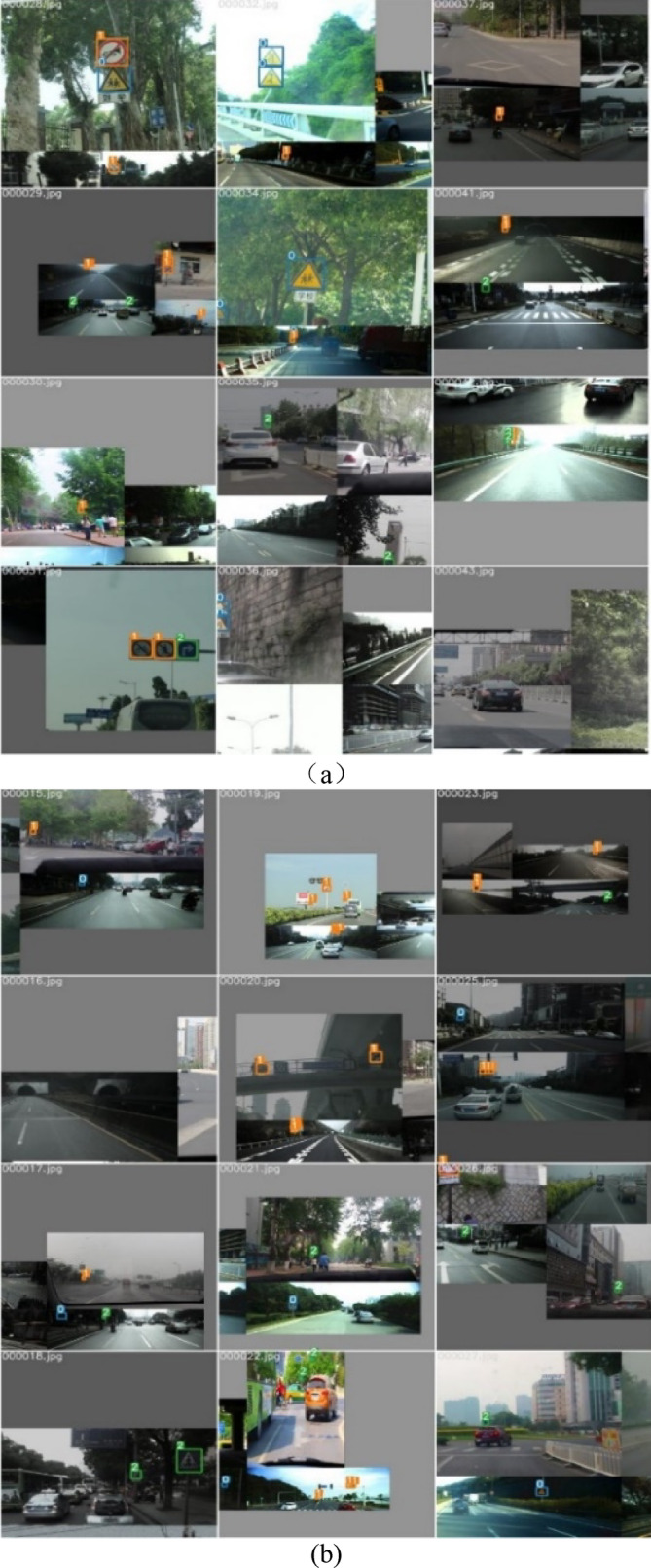

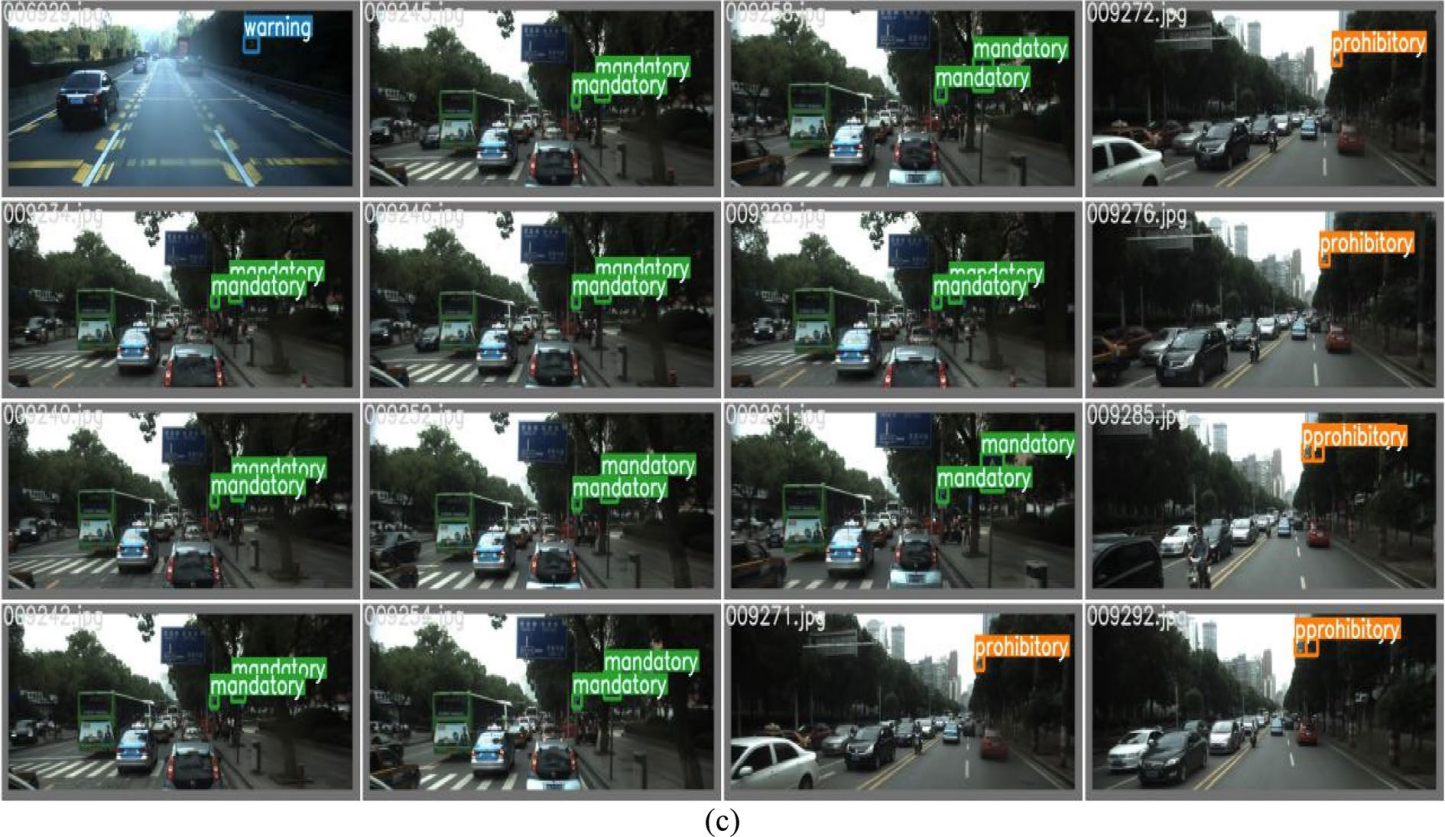
Pretreatment of traffic signs. (**a**) Train-batch1. (**b**) Train-batch2. (**c**) Test-batch-labels.

At the input end of the YOLOv5, the input part can realize data enhancement, adaptive anchor box calculation, and adaptive image scaling. The Mosaic method is used for data enhancement. Data training is performed by randomly selecting several images and then putting them together in one image. At the same time, the image is flipped, and the color gamut is changed, such as the saturation and brightness of the image. The stitching effect in this paper is shown in Fig. 10. The advantage of using the data set enhancement method is that more small targets are included in the randomly scaled images, and the robustness is improved.

## Experiment results and discussion

### Model evaluation indicators

To evaluate the robustness and precision of traffic sign recognition, the evaluation index system of this experiment includes Average Precision (AP), Recall, F1-score, Mean Average Precision (mAP), etc. Among them, the closer the mAP is to 1, the better the overall performance of the model. Since the data set used in the study are divided into three categories: warning, mandatory, and prohibition, the average AP values evaluated are three.

The confusion matrix method is used to better evaluate model performance. The distribution of the confusion matrix is shown in Table 1.

**Table 1:** Confusion matrix.

True value	Predicted value	
	Positive	Negative
Positive	True Positives TP	False Negatives FN
Negative	False Positives FP	True Negatives TN

In Table 1, it is evident that *TP* represents a positive real value and predicted value. In contrast, *TN* is opposite to *TP*, *FP* is a negative real value, while the predicted value is positive, and *FN* is opposite to *FP*. Therefore, the evaluation index formulas in this paper is derived, such as formula (7) to formula (10).

7 $$Precision\;(\% ) = \frac{TP}{{TP + FP}} \times 100\%$$

8 $$Recall\;(\% ) = \frac{TP}{{TP + FN}} \times 100\%$$

9 $$F_{{{1 - }score}} = \frac{2 \times Precision\;(\% ) \times Recall\;(\% )}{{Precision\;(\% ) + Recall\;(\% )}}$$

10 $$mAP = \frac{{\sum\nolimits_{i = 0}^{N = 1} {\int_{0}^{1} {P(R)dR} } }}{N}$$

Among them, the *Precision* (%) represents the accuracy of finding the correct target detection object. The *Recall* (%) represents the ability to find the relevant target detection object. The parameter of the *F*_1*-score*_ combines the output results of *Precision* (%) and *Recall* (%). The value range of the *F*_1*-score*_ is zero to one, when the value of the *F*_1*-score*_ is larger, the output effect is better. Therefore, “zero” refers to the worst performance, and “one” refers to the best performance of the model. The parameter of the *mAP* refers to the average accuracy of detecting *N* classes, and *N* in this paper is one.

### Model training and Verification

#### Super parameter setting

To have a deep understanding of the algorithm, this paper uses the modified pre-training model and YOLOv5s, YOLOv5m, and YOLOv5x for training, respectively, to get the experimental data.

To achieve a better training effect, this paper modifies the threshold of the epoch. When the epochs is 100, 200, and 300, respectively, the experimental data are obtained. Finally, the group with the best data is selected as the final model training data for the model for the traffic sign recognition experiment of the traffic sign recognition system. The adjustment of other super parameters is shown in Table 2.

**Table 2:** Model training super parameter setting.

Type	Learning rate	Momentum	GIOU	CLS	obj	IOU_T	anchor_T
Parameter	0.01	0.937	0.05	0.58	1.00	0.20	4.00

Where, GIOU represents GIOU-Loss confidence loss coefficient, CLS represents CLS loss classification loss coefficient, obj represents obj loss target loss coefficient, IOU_T indicates the IOU threshold of the tag and the anchors IOU training threshold, anchor_T indicates the length h width of the label w/length h of the anchor_A width W_A threshold, i.e. h/h_a, w/w_A must be between (1/2.91, 2.91) anchor multiple thresholds. The following will introduce the result analysis of the epoch when it is set to 100, 200 and 300, etc.

#### Model training

The following will introduce the analysis of the results obtained when the pre-training model is YOLOv5s, YOLOv5m, and YOLOv5x, etc.

##### YOLOV5s

**Fig. 11:**
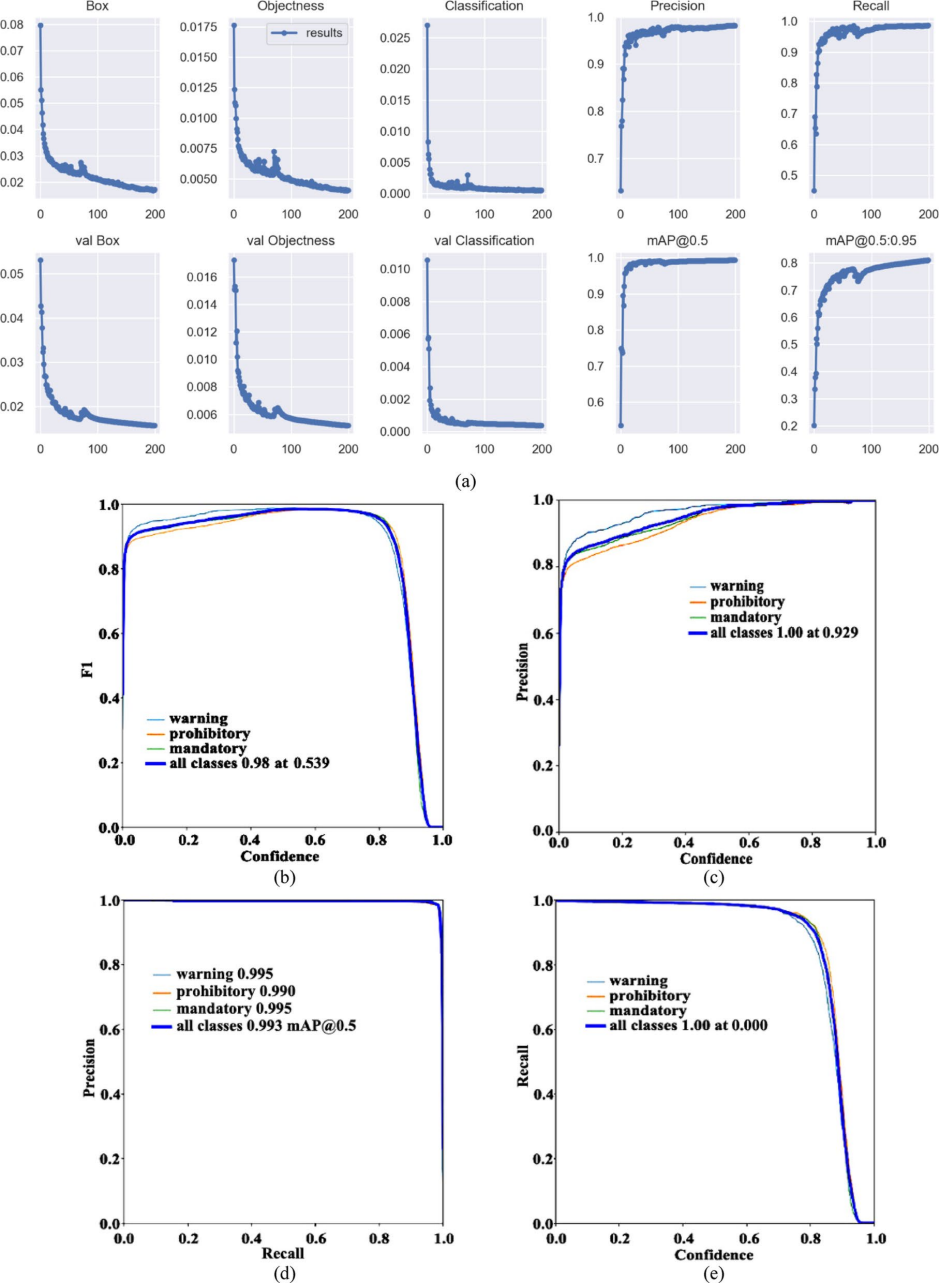

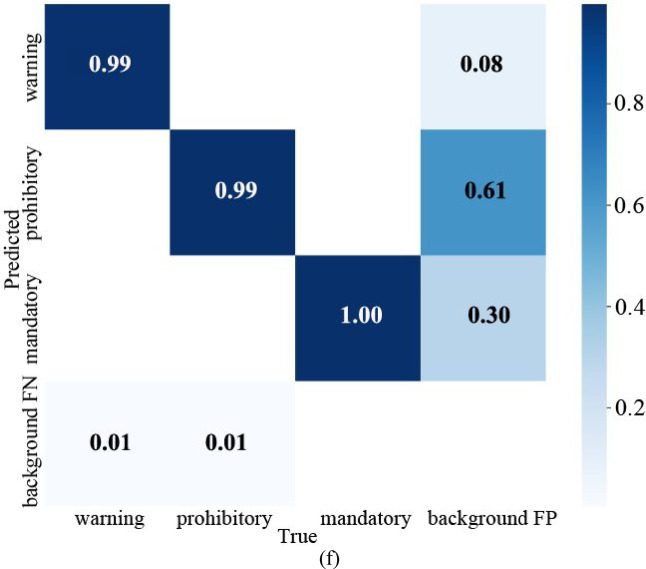
Visual data diagram of yolov5s training. (**a**) Indicator change chart. (**b**) F1_curve. (**c**) P_curve. (**d**) PR_curve. (**e**) R_curve. (**f**) Confusion matrix.

When the pre-training model is YOLOv5s, the model training results, the confusion matrix, and the result graph are shown in Fig. 11.

The parameter mAP@0.5 represents that IOU is set to 0.5, calculates the AP of all pictures in each category, and then averages all categories. The parameter mAP@0.5:0.95 represents the average mAP at different IOU thresholds (from 0.5 to 0.95, in steps of 0.05) (0.5, 0.55, 0.6, 0.65, 0.7, 0.75, 0.8, 0.85, 0.9 and 0.95). According to the Fig. 11a, when the training is about 30 rounds, mAP@0.5 is about 1. When the number of training rounds increases, the mAP@0.5:0.95 has also been improving, indicating that the accuracy of different IOU thresholds is constantly improving, and finally reached 0.8, with high accuracy.

To evaluate the performance metrics of the YOLOv5s model, Fig. 11b–e present the F1_curve, P_curve, PR_curve, and R_curve, respectively. The F1_curve is a measurement standard in classification problems, representing the harmonic mean of precision and recall. Its value ranges from 0 to 1, with 1 being the best and 0 being the worst. From the figure, it can be seen that the F1_curve value eventually reaches 0.98, indicating excellent detection accuracy. The P_curve illustrates the relationship between precision and confidence. As confidence increases, category detection becomes more accurate, with all classification values in the figure ultimately reaching 1.00. The PR_curve reflects the relationship between precision and recall, higher precision often comes at the cost of lower recall. Therefore, it is desirable for the curve to approach the point (1, 1), meaning that the area under the mAP curve should ideally be close to 1. The final values for all classifications reach 0.993, with specific values of 0.995, 0.990, and 0.995 for warning, prohibitory, and mandatory, respectively. The R_curve depicts the relationship between recall and confidence. When confidence is lower, category detection becomes more comprehensive. The figure suggests that the network’s detection is quite comprehensive.

From the confusion matrix in Fig. 11f, it can be seen that the detection rate of warning is 0.99, and the probability of making the second kind of error (not detected, but there is this) is 0.01. The probability of making the first type of error (detected, but without the target) is 0.08. The detection rate of prohibitory is 0.99, the error rate of the second type is 0.01, and the first type is 0.61. The detection rate of mandatory is 1, the error rate of the second type is 0, and the first type is 0.30.

##### YOLOV5m

**Fig. 12:**
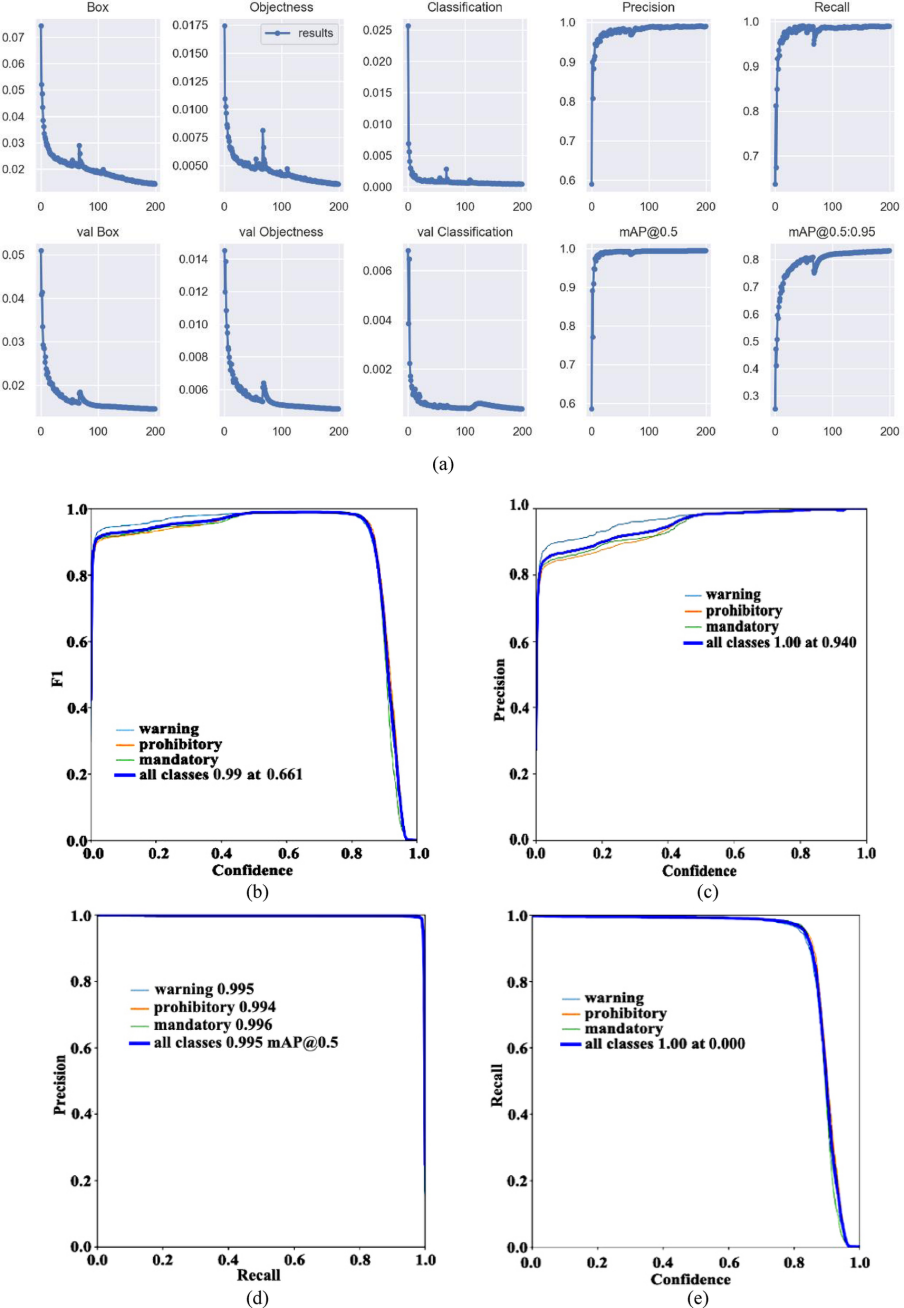

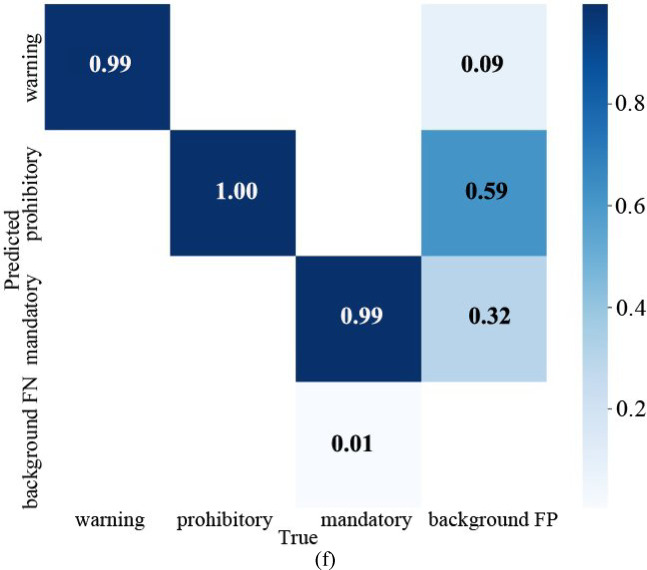
Visual data diagram of yolov5m training. (**a**) Indicator change chart. (**b**) F1_curve. (**c**) P_curve. (**d**) PR_curve. (**e**) R_curve. (**f**) Confusion matrix.

From the above, similarly, the visual data when the pre-training model is YOLOv5m is obtained, as shown in Fig. 12a.

The parameter mAP@0.5 represents that IOU is set to 0.5, calculates the AP of all pictures in each category, and then averages all categories. The parameter mAP@0.5:0.95 represents the average map at different IOU thresholds (from 0.5 to 0.95, in steps of 0.05) (0.5, 0.55, 0.6, 0.65, 0.7, 0.75, 0.8, 0.85, 0.9 and 0.95). According to the Fig. 12a, when the training is about 30 rounds, mAP@0.5 is about 1. When the number of training rounds increases, the mAP@0.5:0.95 has also been improving, indicating that the accuracy of different IOU thresholds is constantly improving, and greater than 0.8, with high accuracy.

To evaluate the performance metrics of the YOLOv5m model, Fig. 12b–e present the F1_curve, P_curve, PR_curve, and R_curve, respectively. From the figure, it can be seen that the F1_curve value eventually reaches 0.99, indicating excellent detection accuracy. As confidence increases, category detection becomes more accurate, with all classification values in the figure ultimately reaching 1.00. The final values for all classifications reach 0.995, with specific values of 0.995, 0.994, and 0.996 for warning, prohibitory, and mandatory, respectively. The figure suggests that the network’s detection is quite comprehensive.

From the confusion matrix in Fig. 12f, we can see that the detection rate of warning is 0.99, while the probability of making the second type of error is 0, and the probability of making the first type of error is 0.09. The detection rate of prohibitory is 1, the second type of error rate is 0, and the first type is 0.59. The detection rate of mandatory is 0.99, the second type of error rate is 0.01, and the first type of error rate is 0.32.

##### YOLOV5x

**Fig. 13:**
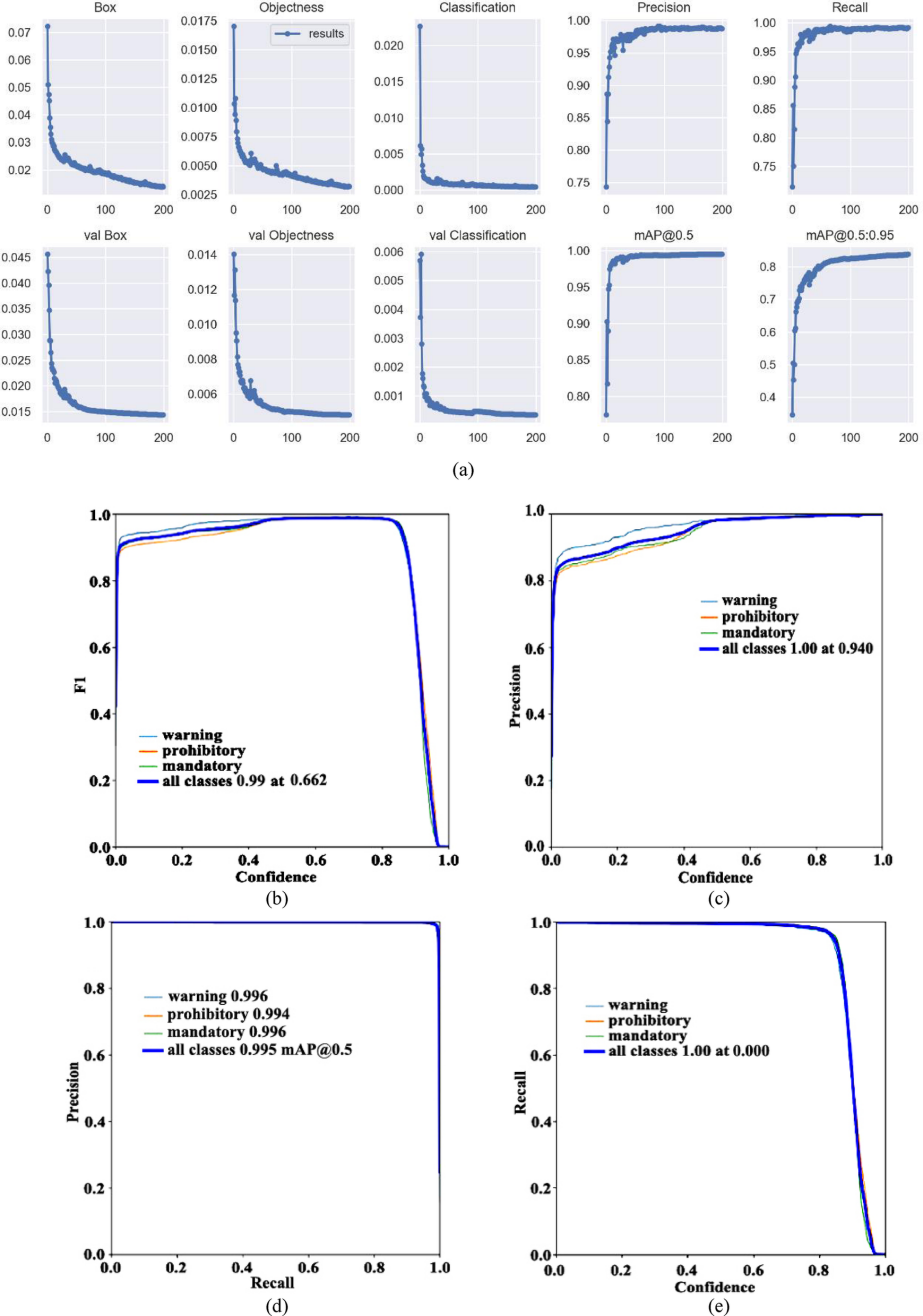

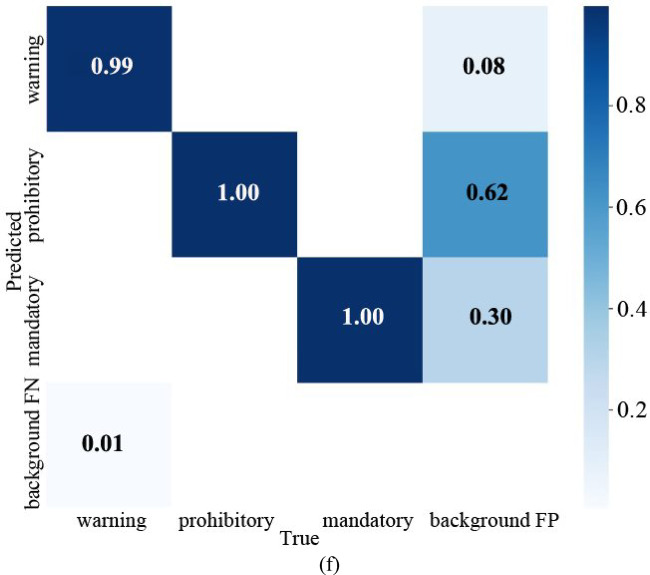
Visual data diagram of yolov5x training. (**a**) Indicator change chart. (**b**) F1_curve. (**c**) P_curve. (**d**) PR_curve. (**e**) R_curve. (**f**) Confusion matrix.

As mentioned above, similarly, the visual data obtained when the pre-training model is YOLOv5x is shown in Fig. 13a.

The parameter mAP@0.5 is that IOU is set to 0.5, calculates the AP of all pictures in each category, and then averages all categories. mAP@0.5:0.95 represents the average map at different IOU thresholds (from 0.5 to 0.95, in steps of 0.05) (0.5, 0.55, 0.6, 0.65, 0.7, 0.75, 0.8, 0.85, 0.9 and 0.95). According to the Fig. 13a, when the training is about 20 rounds, mAP@0.5 is about 1. When the number of training rounds increases, the mAP@0.5:0.95 has also been improving, indicating that the accuracy of different IOU thresholds is constantly improving, and greater than 0.8, with high accuracy.

To evaluate the performance metrics of the YOLOv5x model, Fig. 13b–e present the F1_curve, P_curve, PR_curve, and R_curve, respectively. From the figure, it can be seen that the F1_curve value eventually reaches 0.99, indicating excellent detection accuracy. As confidence increases, category detection becomes more accurate, with all classification values in the figure ultimately reaching 1.00. The final values for all classifications reach 0.995, with specific values of 0.996, 0.994, and 0.996 for warning, prohibitory, and mandatory, respectively. The figure suggests that the network’s detection is quite comprehensive.

From the confusion matrix in Fig. 13f, it can be seen that the warning detection rate is 0.99, the probability of making the second type of detection error is 0.01, and the probability of making the first type of detection error is 0.08. The detection rate of prohibitory is 1, the error rate of the second type is 0, and the first type is 0.62. The detection rate of mandatory is 1, the error rate of the second type is 0, and the first type is 0.30.

At the same time, in order to compare the performance of YOLO with different types of target detection networks, the comparison and verification are carried out on Faster-RCNN, SSD, EfficientDetD0 and EfficientDetD2, respectively. The results are shown in Table 3.

**Table 3:** Training data comparison.

Model	Performance index				
	Size (pixels)	mAP@0.5:0.95 (%)	mAP@0.5 (%)	Speed (ms)	Params (M)
YOLOv5s	640	81.0	99.3	98	14.1
YOLOv5m	640	83.2	99.4	224	40.8
YOLOv5x	640	83.8	99.5	766	166
Faster-RCNN	640	45.38	84.7	128.4	41.8
SSD	640	49.41	81.7	48.6	36
EfficientDet D0	640	59.22	76.3	42	4
EfficientDet D2	640	76.94	96.6	75.5	8.1

Compared with more advanced single-stage detectors or improved network structures, Faster-RCNN has a moderate level of accuracy, while SSD (VGG19) has a higher level of accuracy compared to Faster-RCNN, mAP@0.5: 0.95 is slightly higher, but mAP@0.5 slightly lower. The overall difference between Faster-RCNN and SSD (VGG19) is not significant. EfficientDetD0 shows a significant improvement in accuracy compared to traditional SSD/Faster-RCNN at a stricter ratio of mAP@0.5:0.95 (59.22% > 49.41% or 45.38%), but its absolute value at mAP@0.5 is slightly lower than the former because it performs better on small targets and stricter boundaries. The result of EfficientDetD2 shows a significant improvement compared to the previous models, indicating a more accurate positioning accuracy. YOLOv5 series (s/m/x) mAP@0.5 0.95 is between 81.0% and 83.8%, mAP@0.5 is up to 99.3–99.5%, very close to the perfect score, indicating that YOLOv5 has almost achieved “ceiling level” accuracy in this traffic sign detection task.

In terms of inference speed and model size, Faster-RCNN has a processing speed of 128.4 ms/sheet and a parameter count of 41.8 M. The speed is moderate and the parameter count is not small. The SSD has the speed of 48.6 m/s per sheet and runs very fast with a parameter count of 36 M. Its advantage is good real-time performance, reaching up to 21FPS, but the problem of missed detections is obvious. EfficientDet D0 has the speed of 42 ms/sheet (24FPS) and only 4 M parameters, making it very lightweight and achieving a balance between low model size, observable speed, and good accuracy. The speed of EfficientDet D2 is 75.50 ms/sheet (13FPS), with a parameter of 8.1 M. Compared with EfficientDet D0, the accuracy is greatly improved, but the speed slows down and the parameter count doubles. The YOLOv5 series (s/m/x) shows that as the model grows, the inference speed significantly decreases and the number of parameters sharply increases, but the accuracy also increases from 81% to 83.8% (mAP@0.5: 0.95) slightly improved. If high real-time performance is required and a slight loss of accuracy can be tolerated, v5s can be selected. For the highest accuracy and strong computing power support, v5x can be used.

In summary, Faster-RCNN and SSD are among the earlier detectors with advantages in certain aspects such as high recall or high speed, but their overall performance has been surpassed by new methods. EfficientDet has achieved significant accuracy improvement while maintaining model lightweight through more advanced backbone networks, BiFPN, multi-scale feature fusion, and other techniques. EfficientDet D0 and EfficientDet D2 are suitable for high-precision detection on mobile devices or devices with limited computing power. YOLOv5 performs extremely well in tasks such as traffic sign detection, providing multiple scale versions such as s/m/x, which can be flexibly selected according to computing power and demand, and its missed and false detections are almost zero.

#### Performance indicators and statistical verification

In order to compare the performance of YOLOv5(YOLOv5s) and different object detection models on the mAP, 30 sampling evaluations were conducted from the complete test set. In each evaluation, 20% of the test set is randomly selected as the data for this evaluation, and the mAP of each model is calculated, and descriptive statistics were summarized and calculated. Table 4 displays information such as sample number (count), mean, standard deviation, and quantile for each model.

**Table 4:** Model performance evaluation.

Model	Count	Mean	Std	Min	Max	
YOLOv5	30	99.39	0.14	99.21	99.60	
Faster-RCNN	30	84.70	0.21	84.46	85.10	
SSD	30	81.55	0.14	81.34	81.76	
EfficientDet D0	30	76.26	0.15	76.04	76.56	
EfficientDet D2	30	96.55	0.19	96.27	96.91	

From the above data, it can be seen that there are significant differences in the performance of each model on mAP, with Yolov5 having the highest mean, followed by EfficientDet D2, and then Faster-RCNN, SSD, and EfficientDet D0.

Subsequently, normality and homogeneity of variance tests were conducted on each model. Before conducting statistical inference, the mAP data of each model were first tested to ensure that they meet the prerequisites of parameter statistical methods. The Shapiro–Wilk test was used to examine the data of each model, and the results showed that the P-values of all models were greater than 0.05, as shown in Table 5. The analysis results indicate that the mAP data of each model approximately follows a normal distribution.

**Table 5:** Model normality test P-value.

Model	YOLOv5	Faster-RCNN	SSD	EfficientDet D0	EfficientDet D2
Normality P-value	0.2653	0.4207	0.6771	0.7715	0.8620

Subsequently, Levene test was used to check whether the variances of each group of data were equal, and the P-value was 0.6381 (greater than 0.05), indicating that there was no significant difference in variance between the models. In summary, both prerequisites have been met, indicating that one-way analysis of variance (ANOVA) can be used for subsequent analysis. When both normality and homogeneity of variance are satisfied, ANOVA test is used to compare the differences in mAP mean between different models. The test results showed that the P-value of ANOVA was less than 0.0001, indicating that at least one group of models had significant differences in average mAP compared to the other groups.

In order to further clarify the differences between the models, Tukey HSD post hoc tests were used to perform paired comparisons of the models. The test results show that there are significant differences between the models, and the specific comparison results are shown in Table 6.

**Table 6:** Statistical analysis results.

Group1	Group2	Mean difference	P-adjust	Lower bound of confidence interval	Upper bound of confidence interval	Reject the null hypothesis
YOLOv5	Faster-RCNN	− 14.6891	0.0	− 14.9027	− 14.4756	True
YOLOv5	SSD	− 17.8354	0.0	− 18.0489	− 17.6218	True
YOLOv5	EfficientDet D0	− 23.1251	0.0	− 23.3386	− 22.9115	True
YOLOv5	EfficientDet D2	− 2.8378	0.0	− 3.0514	− 2.6243	True
Faster-RCNN	SSD	− 3.1462	0.0	− 3.3598	− 2.9327	True
Faster- RCNN	EfficientDet D0	− 8.4359	0.0	− 8.6495	− 8.2224	True
Faster- RCNN	EfficientDet D2	11.8513	0.0	11.6377	12.0648	True
SSD	EfficientDet D0	− 5.2897	0.0	− 5.5033	− 5.0762	True
SSD	EfficientDet D2	14.9975	0.0	14.7840	15.2111	True
EfficientDet D0	EfficientDet D2	20.2872	0.0	20.0737	20.5008	True

According to Table 6, the mAP value of YOLOv5 is significantly higher than that of Faster-RCNN, SSD, and EfficientDet D0 models. In comparison with the EfficientDet D2 model, although the mAP difference between the two is relatively small, after rigorous statistical analysis, the performance advantage of Yolov5 still reaches a significant level. This result fully demonstrates the outstanding performance of YOLOv5 in object detection tasks. Secondly, a comparative analysis was conducted on the performance of the EfficientDet D2 model with other models. The results showed that the mAP value of EfficientDet D2 was second only to Yolov5, but significantly better than Faster-RCNN, SSD, and EfficientDet D0 models. In addition, detailed comparisons were made between the performances of other models. Research has found that Faster-RCNN performs better than SSD and EfficientDet D0 models, and the mAP value of the SSD model is higher than that of the EfficientDet D0 model. This result clearly reveals the performance differences and hierarchical relationships among different models in object detection tasks.

In order to evaluate the performance of each model more scientifically, Tukey HSD (Honest Significant Difference) method was used for multiple comparative analysis. Based on the comparison results of Tukey HSD, the ranking of model performance was obtained: YOLOv5 > EfficientDet D2 > Faster-RCNN > SSD > EfficientDet D0. In summary, this study comprehensively evaluated the performance of five mainstream object detection models in object detection tasks by comparing their mAP values. Among them, the YOLOv5 model exhibits the most superior performance, followed by the EfficientDet D2, Faster-RCNN, SSD, and EfficientDet D0 models in that order.

#### Real-world validation

**Fig. 14:**
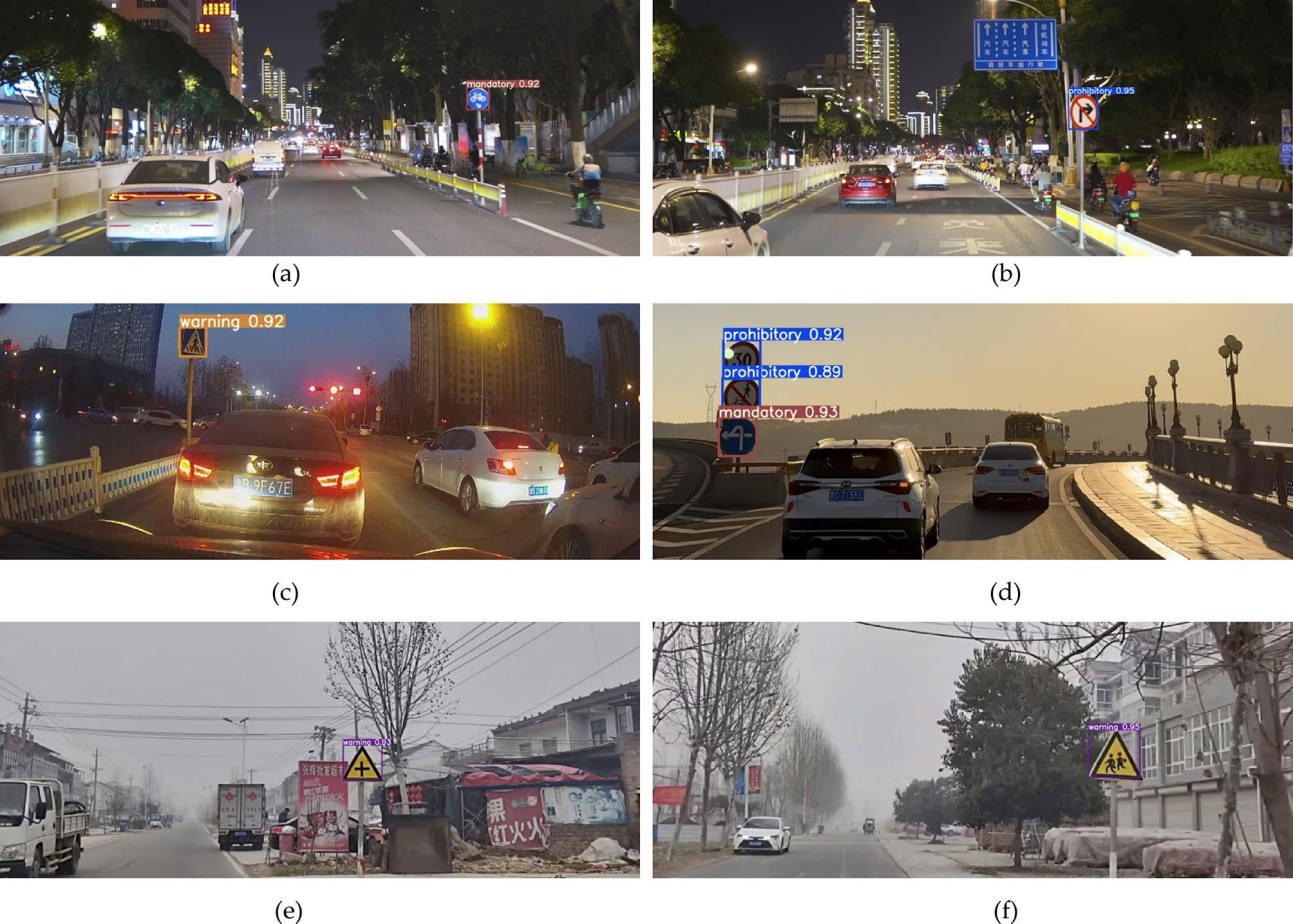
Real-world validation. (**a**) Small sample 1. (**b**) Small sample 2. (**c**) Backlit sample 1. (**d**) Backlit sample 2. (**e**) Foggy scenes sample 1. (**f**) Foggy scenes sample 2.

For difficult to detect target samples such as small samples, backlit and foggy scenes, the constructed networks can achieve effective detection with detection confidence levels exceeding 0.90, fully verifying the excellent performance of the network in complex environments, as shown in Fig. 14. Firstly, small sample detection is a major challenge in object detection tasks, especially when the target occupies a small pixel area in the image, traditional methods often struggle to accurately locate and recognize it. The experimental results show that even if the target size is small, the network can still maintain a high detection confidence (> 0.92), fully reflecting its efficient ability to capture detailed features. Secondly, under backlight conditions, the contrast between the target and background is low, and traditional methods are prone to missed or false detections due to uneven lighting. In backlit scenarios, the network can still stably output high confidence detection results (> 0.89), demonstrating its strong adaptability to changes in lighting conditions. In foggy environments, the constructed network still maintains a high detection confidence level (> 0.93), fully demonstrating its robustness to complex environments. In summary, the constructed network exhibits good detection performance in small sample, backlit, and foggy scenes. This result not only verifies the strong generalization ability of the network in diverse scenarios, but also highlights its high robustness in complex environments.

## Conclusions

This study is based on the YOLOv5 model and systematically investigates and experimentally verifies the task of traffic sign detection. By constructing the CCTSDB dataset and combining the k-means++ algorithm to optimize anchor boxes, Mosaic data augmentation techniques, and various hyperparameter adjustment strategies, the detection performance of the model in complex scenes has been significantly improved.

The experimental results show that the YOLOv5 series models perform well in traffic sign detection tasks, especially in small sample, backlit, and foggy scenes, with detection confidence levels exceeding 0.90, verifying the strong generalization ability and robustness of the models in diverse environments. By using the k-means++ algorithm for clustering analysis of the dataset, the distribution of anchor boxes was optimized, significantly improving the model’s ability to detect small targets. Experiments have shown that the k-means++ algorithm has significant advantages in reducing computational complexity and accelerating convergence, with an average IoU of 77.55%, which is better than the traditional k-means algorithm’s 75.95%. Through Mosaic data augmentation technology, the diversity of training data has been enriched, the adaptability of the model to complex scenes has been improved, and the detection accuracy of the model has been significantly enhanced. Through comparative experiments on YOLOv5s, YOLOv5m, and YOLOv5x models of different scales, it was found that as the model size increased, the detection accuracy gradually improved, but the inference speed decreased. YOLOv5 series (s/m/x) mAP@0.5:0.95 is between 81.0% and 83.8%, mAP@0.5 is between 99.3% and 99.5%, indicating that YOLOv5 has almost achieved “ceiling level” accuracy in this traffic sign detection task. Through comparative experiments with mainstream object detection models such as Faster-RCNN, SSD, EfficientDet D0, and EfficientDet D2, the YOLOv5s model demonstrates superior detection accuracy (mAP@0.5:0.95) and inference speed. Through Tukey HSD multiple comparison analysis, the outstanding performance of YOLOv5s in object detection tasks was further verified. In practical scenario verification, the network constructed in this study demonstrated excellent detection performance in small sample, backlit, and foggy scenarios, with detection confidence levels exceeding 0.90. Especially in complex environments such as backlighting and foggy weather, the model can still stably output high confidence detection results, fully demonstrating its high adaptability to changes in lighting and complex environments.

The next step is to further optimize the model structure and reduce computational complexity while ensuring detection accuracy, in order to adapt to embedded devices and mobile applications. Meanwhile, more advanced data augmentation techniques can also be introduced to further enhance the model’s generalization ability.

## Electronic supplementary material

Below is the link to the electronic supplementary material.

Supplementary Material 1

Supplementary Material 2

## Data Availability

CCTSDB data set can be downloaded on “https://github.com/csust7zhangjm/CCTSDB”.
