# Supplementary material for: Efficient traffic sign recognition using YOLO for intelligent transport systems
Source: Sci Rep. 2025 Apr 21;15:13657. doi: 10.1038/s41598-025-98111-y (PMC12009977; doi:10.1038/s41598-025-98111-y)
Supplement: Supplementary file 2 — Supplementary Material 2 [file 41598_2025_98111_MOESM2_ESM.docx]

**Test of Traffic Sign Detection through Driving Recorder Videos**

This study employs the YOLO network to conduct target detection analysis on driving recorder videos, focusing specifically on traffic signs categorized as Warning, Prohibitory, and Mandatory. Through detection data collected under varying time segments and distances, the study systematically evaluates the network's detection accuracy and stability in complex traffic scenarios.

Typical urban road driving recorder videos were selected as experimental materials, featuring multiple traffic scene transitions and target objects at varying distances. The YOLOv5 network architecture was utilized to achieve real-time detection of traffic signs. Specific detection results are as follows:

(1) Prohibitory sign test (6-8 seconds)

During the initial video segment (6-8 seconds), the detection scenario presents typical long-distance target characteristics. When the target object is at a considerable distance, the YOLO network successfully identifies Prohibitory sign with a confidence score of 0.84. As the vehicle continues to approach, the target features become clearer, and the network's confidence score increases to 0.89. With further reduction in detection distance, the confidence score peaks at 0.93, as shown in Figure 1.


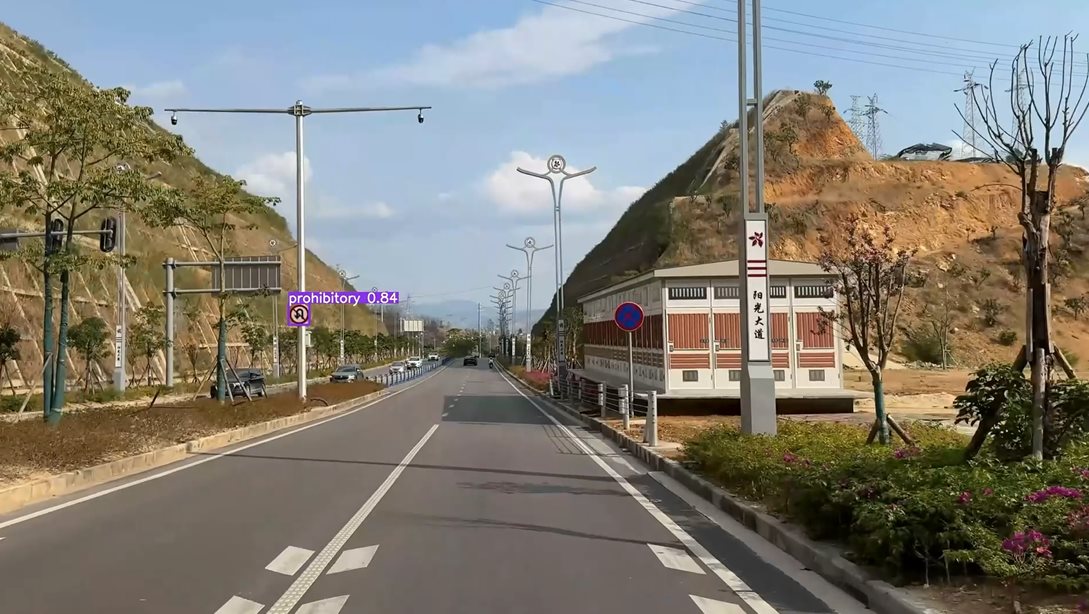


(a)


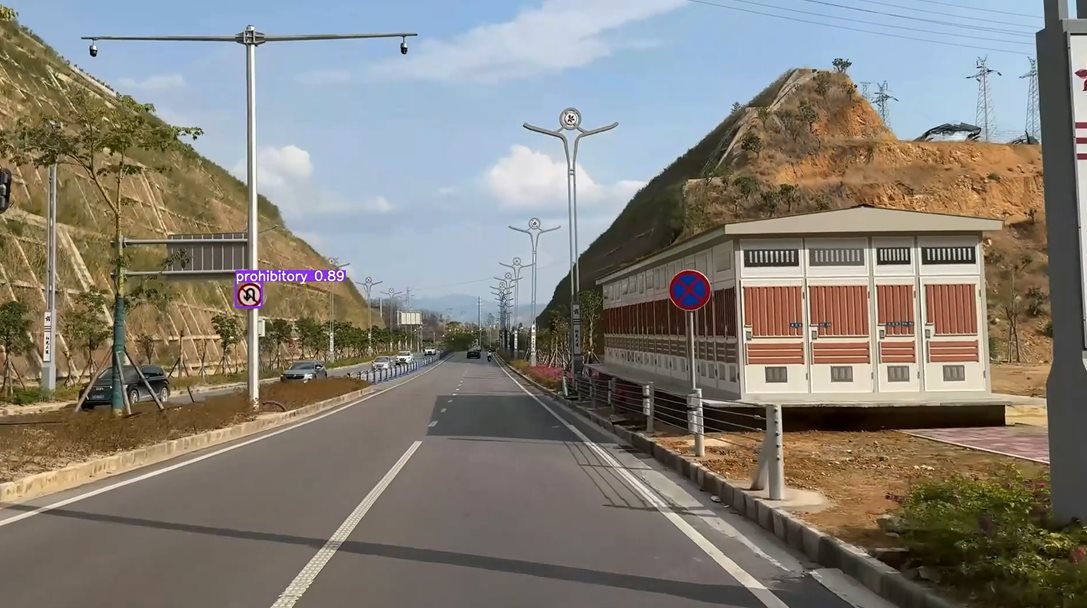


(b)


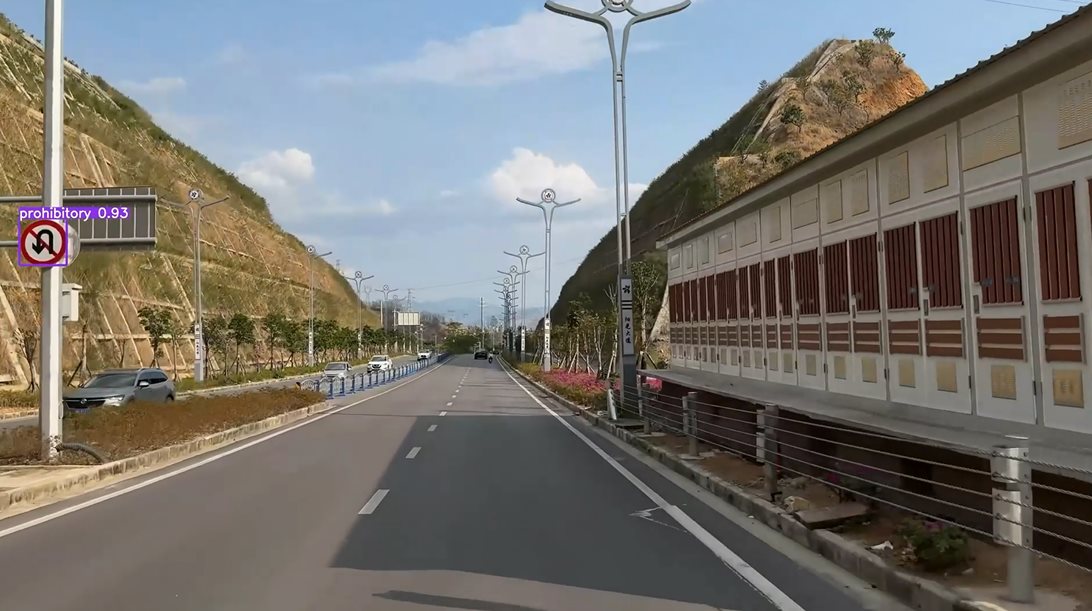


(c)

Figure 1 Prohibitory sign test

(2) Dual-target sign test (18-21 seconds)

This segment features a complex scenario with both Warning and Prohibitory signs present. At a longer distance, the network achieves a confidence score of 0.87 for the Prohibitory sign and 0.73 for the Warning sign. As the vehicle approaches, the confidence scores for both signs increase to 0.89 and 0.82, respectively. With further reduction in detection distance, the scores rise to 0.92 and 0.90. Notably, during dual-target detection, the network maintains stable recognition accuracy without target confusion, attributed to its anchor box optimization mechanism, as shown in Figure 2.


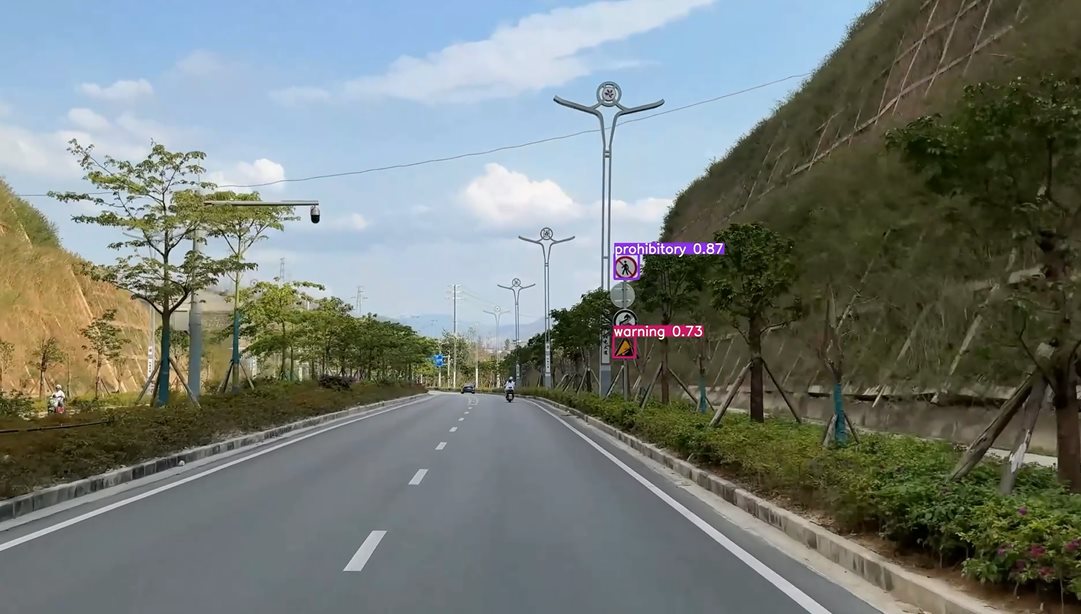


(a)


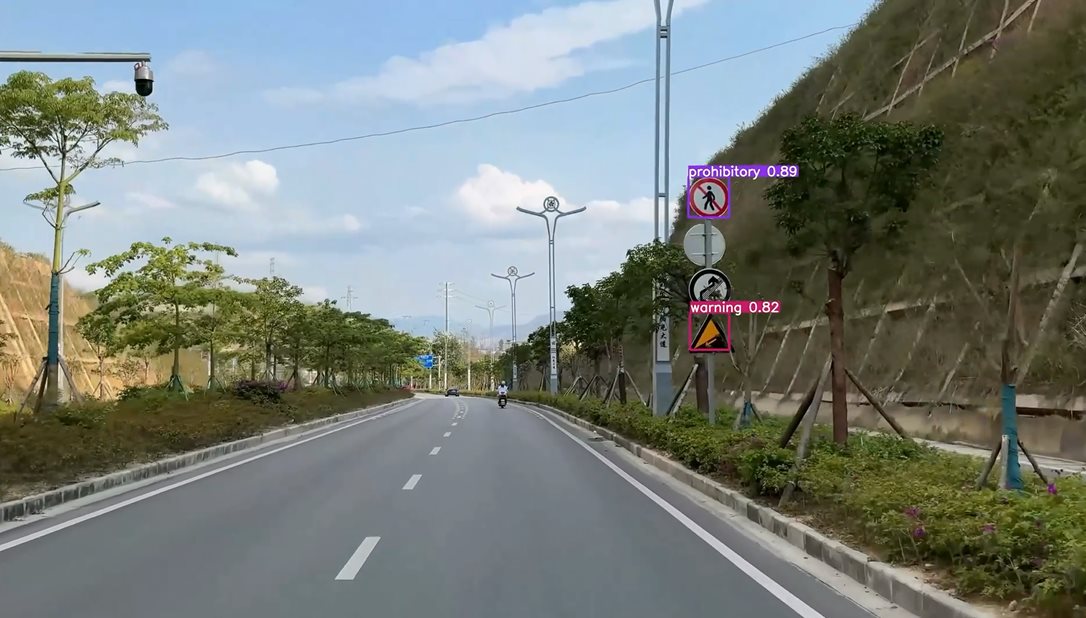


(b)


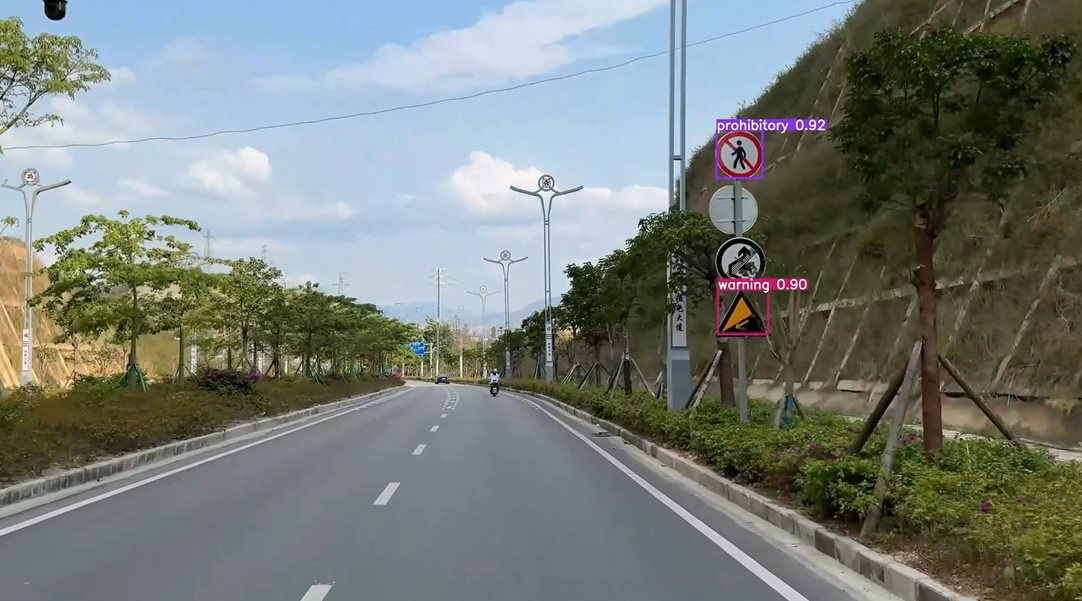


(c)

Figure 2 Dual-target sign test

(3) Warning sign test (29-32 seconds)

This phase primarily tests Warning sign detection. At a longer distance, only the Warning sign is detected with a confidence score of 0.62. As the vehicle continues, the network assigns a confidence score of 0.87 to this Warning sign. With further approach, a confidence score of 0.93 to this sign. This experimental result demonstrates the YOLO network's real-time target tracking capability and robust dynamic feature extraction and recognition abilities, as shown in Figure 3.


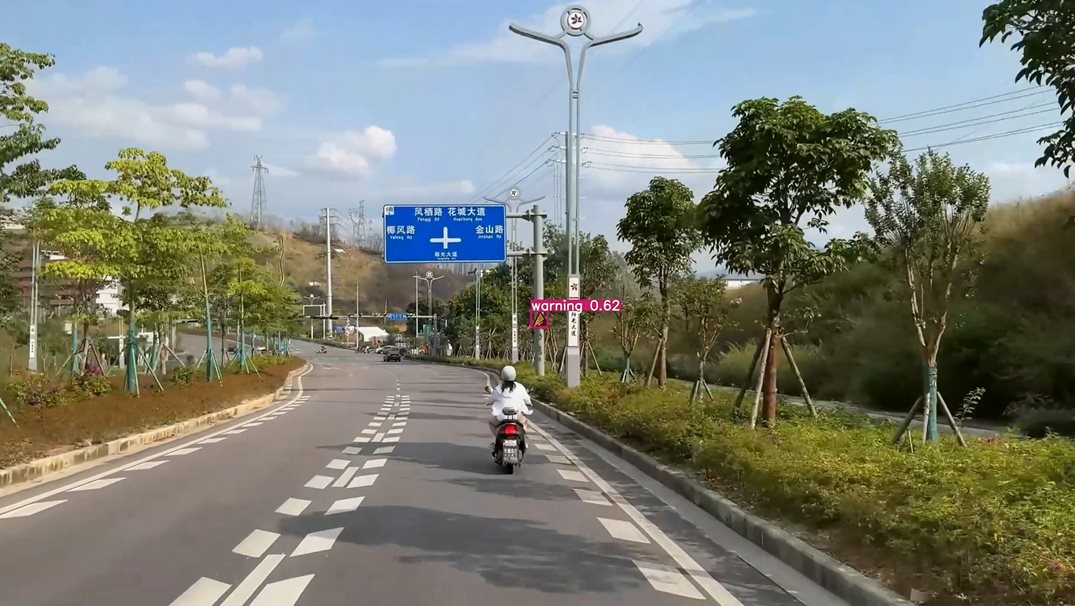


(a)


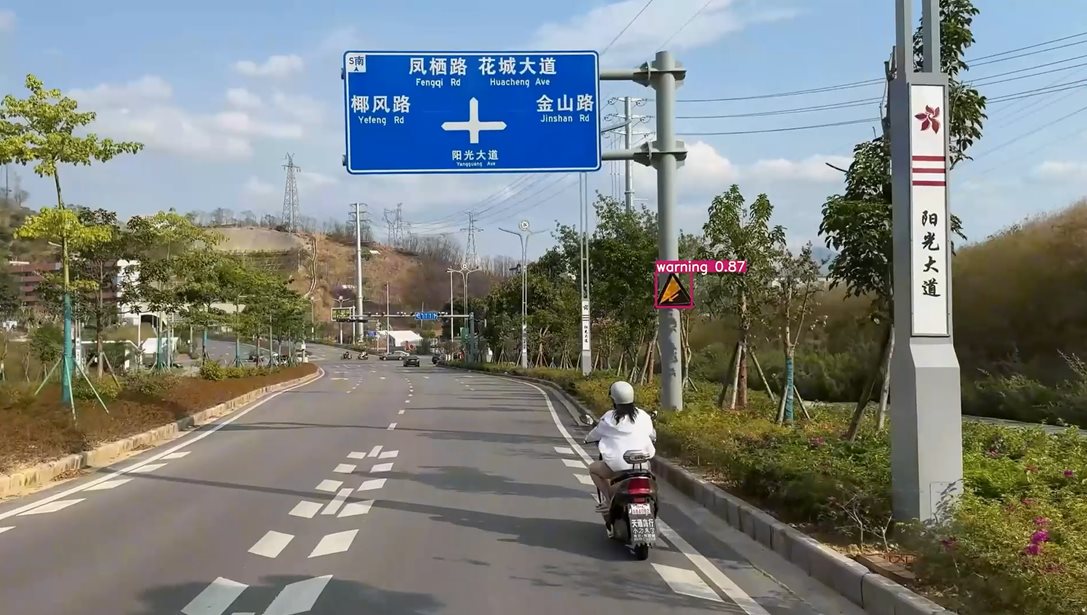


(b)


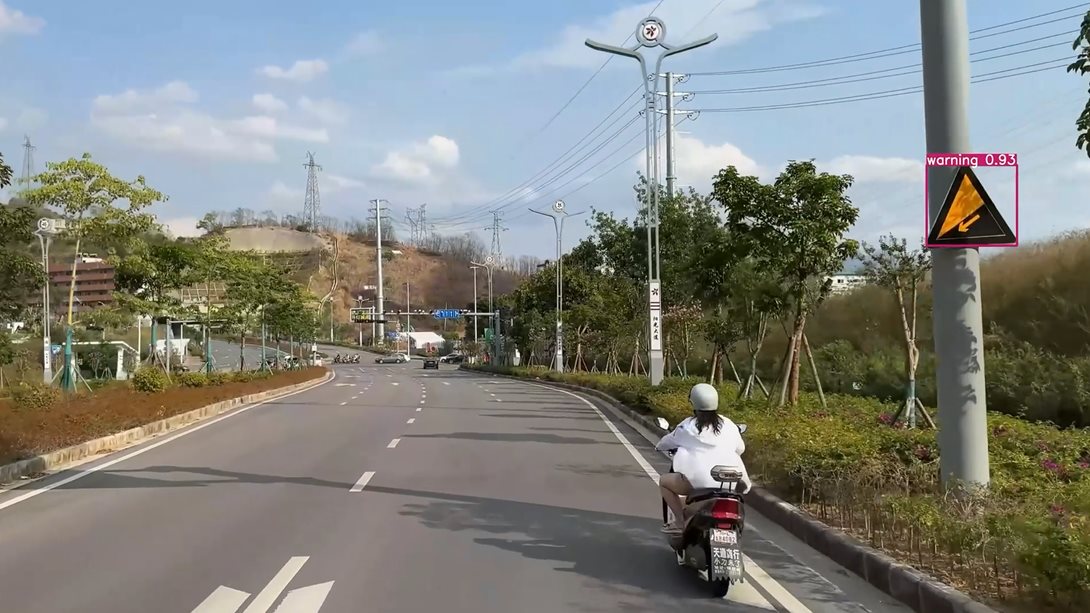


(c)

Figure 3 Warning sign test

(4) Multi-target sign test (54-56 seconds)

This segment presents the most challenging multi-target detection scenario, involving five Prohibitory signs (four primary targets + one distant small target). During initial detection, the four primary Prohibitory targets exhibit confidence scores of 0.82, 0.90, 0.90, and 0.88, respectively. As the vehicle approaches, the confidence scores for the four primary targets increase to 0.89, 0.92, 0.91, and 0.92, the confidence scores of the small target is 0.58. At closer detection distances, the four primary targets stabilize at confidence scores of 0.90, 0.94, 0.93, and 0.94, while the small target achieves 0.70, as shown in Figure 4. This experiment validates the YOLO network's robustness in multi-target scenarios and its effective handling of target overlap issues.

Across all detection segments, the YOLO network demonstrates exceptional target recognition capabilities. Within the detection range, Prohibitory signs achieve an average confidence score of 0.89, while Warning signs reach 0.82. Notably, at close detection distances, both sign types exceed 0.90 confidence, meeting practical traffic monitoring precision requirements.


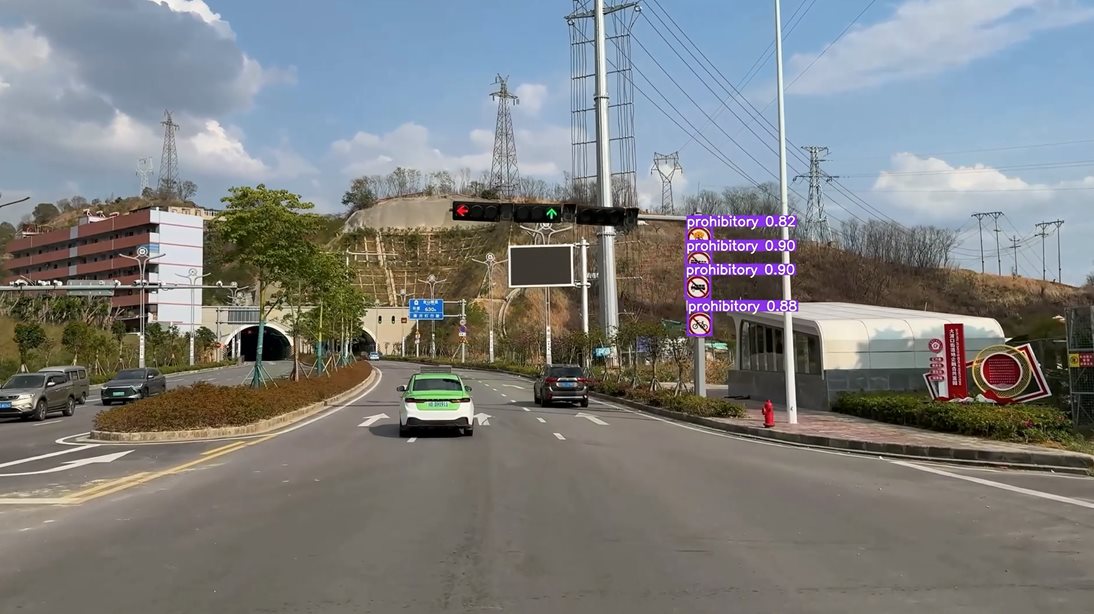


(a)


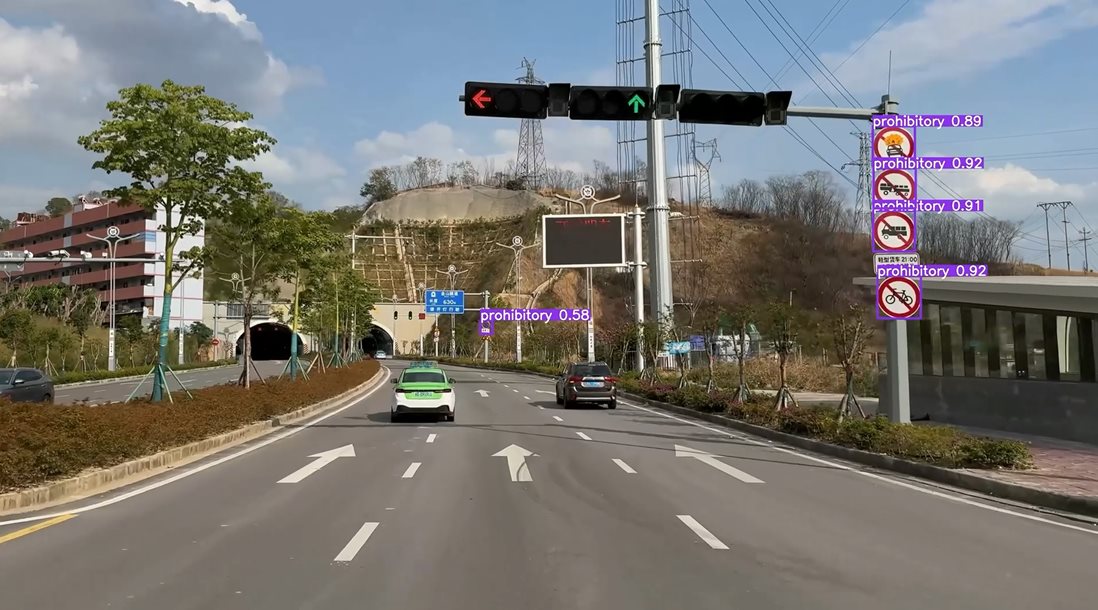


(b)


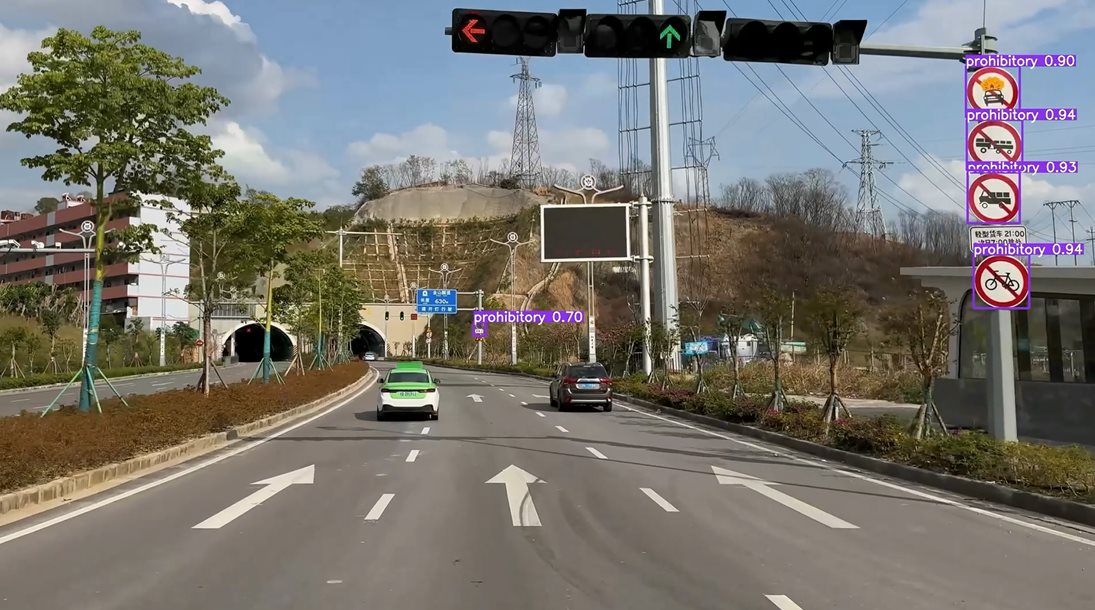


(c)

Figure 4 Multi-target sign test
